# Author Correction: Molecular control via dynamic bonding enables material responsiveness in additively manufactured metallo-polyelectrolytes

**DOI:** 10.1038/s41467-024-52457-5

**Published:** 2024-09-16

**Authors:** Seola Lee, Pierre J. Walker, Seneca J. Velling, Amylynn Chen, Zane W. Taylor, Cyrus J.B.M Fiori, Vatsa Gandhi, Zhen-Gang Wang, Julia R. Greer

**Affiliations:** 1 Division of Engineering and Applied Science, California Institute of Technology, 1200 California Boulevard, Pasadena, 91125 CA USA; 2 Division of Chemistry and Chemical Engineering, California Institute of Technology, 1200 California Boulevard, Pasadena, 91125 CA USA; 3 Kavli Nanoscience Institute, California Institute of Technology, 1200 California Boulevard, Pasadena, 91125 CA USA

**Keywords:** Gels and hydrogels, Coarse-grained models, Polymers, Mechanical properties

Correction to: *Nature Communications* 10.1038/s41467-024-50860-6, published online 10 August 2024

In the Acknowledgements section, the following sentence, ‘The authors gratefully acknowledge the financial support from the Center for Autonomous Systems and Technologies (CAST) at Caltech, the Institute for Collaborative Biotechnologies from the Army Research Office (ARO), and the Schwartz/Reisman Collaborative Science Program from the Schwartz/Reisman Foundation’ should have read, ‘The authors gratefully acknowledge the financial support from the Center for Autonomous Systems and Technologies (CAST) at Caltech and the Schwartz/Reisman Collaborative Science Program from the Schwartz/Reisman Foundation. Research was also sponsored, in part, by the U.S. Army Research Office and accomplished under contract W911NF-19-D-0001 for the Institute for Collaborative Biotechnologies’. The original article has been corrected.

