# Supplementary material for: Molecular control via dynamic bonding enables material responsiveness in additively manufactured metallo-polyelectrolytes
Source: Nat Commun. 2024 Aug 10;15:6850. doi: 10.1038/s41467-024-50860-6 (PMC11316739; doi:10.1038/s41467-024-50860-6)
Supplement: Supplementary file 1 — Supplementary Information [file 41467_2024_50860_MOESM1_ESM.pdf]

Supplementary Information: Molecular Control via Dynamic Bonding Enables Material Responsiveness in Additively Manufactured Metallo-Polyelectrolytes

Seola Lee[1*†], Pierre J. Walker[2†], Seneca J. Velling[1], Amylynn Chen[1], Zane W.Taylor[1], Cyrus J.B.M Fiori[2], Vatsa Gandhi[1,3], Zhen-Gang Wang[2] and Julia R. Greer[1,3]

[1]Division of Engineering and Science, California Institute of Technology, 1200 California Boulevard, Pasadena, 91125, CA, USA.
[2]Division of Chemistry and Chemical Engineering, California Institute of Technology, 1200 California Boulevard, Pasadena, 91125, CA, USA.
[3]Kavli Nanoscience Institute, California Institute of Technology, 1200 California Boulevard, Pasadena, 91125, CA, USA.

*Corresponding author(s). E-mail(s): seolalee@caltech.edu;
†These authors contributed equally to this work.

Supplementary Information Table of Contents

Supplementary Figures

1 Schematic of Liquid Crystal Display (LCD) vat photopolymerization (VP) process. 4
2 Mass tracking of additively manufactured MPEC. 4
3 X-Ray Fluorescence mapping of additively manufactured MPEC 5
4 Mooney-Rivlin plot of the stress-strain curves. 6
5 Fraction of inter-crosslinked ions during deformation. 7
6 Fracture set-up and analysis. 8
7 Stress-strain curves for un-notched and notched MPEC for fracture experiment. 9
8 Plateau modulus of Additively Manufactured MPEC. 10
9 Confocal Microscope Images of Additively Manufactured MPEC. 11
10 pH-dependent opacity and microstructure of Additively manufactured MPEC. 12
11 MPEC swelling and dissolution progress. 13
12 Stress relaxation and recovery of MPECs. 14
13 Representative X-ray Diffraction (XRD) pattern of characteristic MPEC gel. 15
14 Experimental validation of the phase diagram for Ca-MPEC . . 16
15 Experimental validation of the phase diagram of Al-MPEC . . 17
16 Additional experimental validation of the constructed phase diagram for Al-MPEC 18
17 UV-Visible photospectroscopy of relevant, transition metals . . 19
18 ^{57}Co-Moessbauer spectrometry of Fe(III) gel phase and frozen resin (solution) phase 19
19 Near edge X-ray absorption (XANES) spectra of Zn^{2+} and Ni^{2+} 20

Supplementary Tables

1 MPEC photoresin composition. 21
2 LCD printing and post-processing parameters. 22
3 pH measurement of photoresin. 22
4 XRF Counts in constant volume mapping volume across cation and pH ranges tested. 23

Supplementary Discussions

1 Binding Energy of Metal-Acetate Complexes 24
1.1 Binding energy 24
1.2 Molecular orbitals 26

2 Impact of metal ion identity 27
2.1 Theoretical predictions 27
2.2 Experimental observations 29

3 Modified Henderson–Hasselbach Equation 31
3.1 Interplay between pH and metal valency 34

4 Vibrational Spectroscopy for Study of Carboxyl/Carboxylate Association 35
4.1 Carboxylate Metal Coordination 35
4.1.1 Quantum DFT Simulation of IR Spectra for Metal-Carboxylate Coordination 37
4.2 Effect of pH on Hydrogen Bonding in Poly(acrylic acid) Gels . 38

5 Mean-field theory of multivalent ions and polyelectrolyte systems 42
5.1 Excluded Volume Effects 42
5.2 Electrostatic Effects 44
5.3 Association Effects 45
5.4 Definition of phase equilibrium 48
5.5 Determining fraction of interchain crosslinked sites 49

6 Thermogravimetric Analysis of MPEC Gels 50

7 Solvent effect on MPEC gels 53
7.1 Experimental results 53
7.1.1 Effect of Co-solvent on Mechanical Response 53
7.1.2 Effect of Co-Solvent on Evaporative Endotherm 54
7.2 Theoretical results 56

8 Viscoelastic MPECs for controlling mechanical deformation 58
8.1 Strain-rate dependent buckling 58

Supplementary Figures

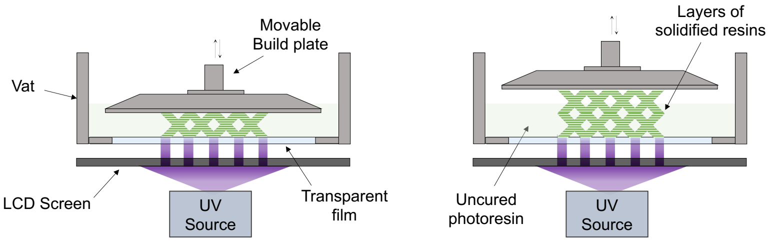

Supplementary Fig. 1: **Schematic of Liquid Crystal Display (LCD) vat photopolymerization (VP) process.** Free-radical photopolymerization reaction is initiated by projecting UV light into a liquid photoresin in the bath. A layer-by-layer printing is processed to produce solidified parts

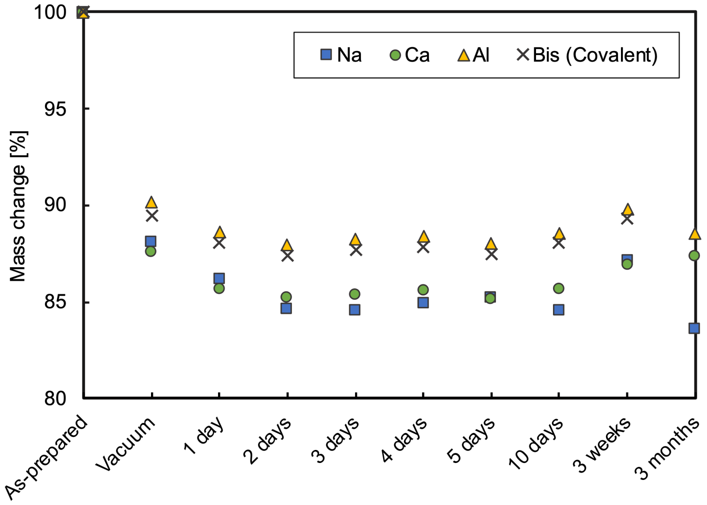

Supplementary Fig. 2: **Mass tracking of additively manufactured MPEC.** Mass change of 3D-printed MPEC with the dimension of 30 $\times$ 6 $\times$ 1.5mm was measured as $\Delta m\% = (m_i - m_{equ})(m_i) \times 100$ where m_i is the initial mass of as-printed MPEC and m_{equ} is the equilibrated mass of MPEC samples after vacuum drying at 20°C and equilibrating in ambient environment. Regardless of pH and metal ions, all samples exhibit high longevity and stability in the ambient conditions.

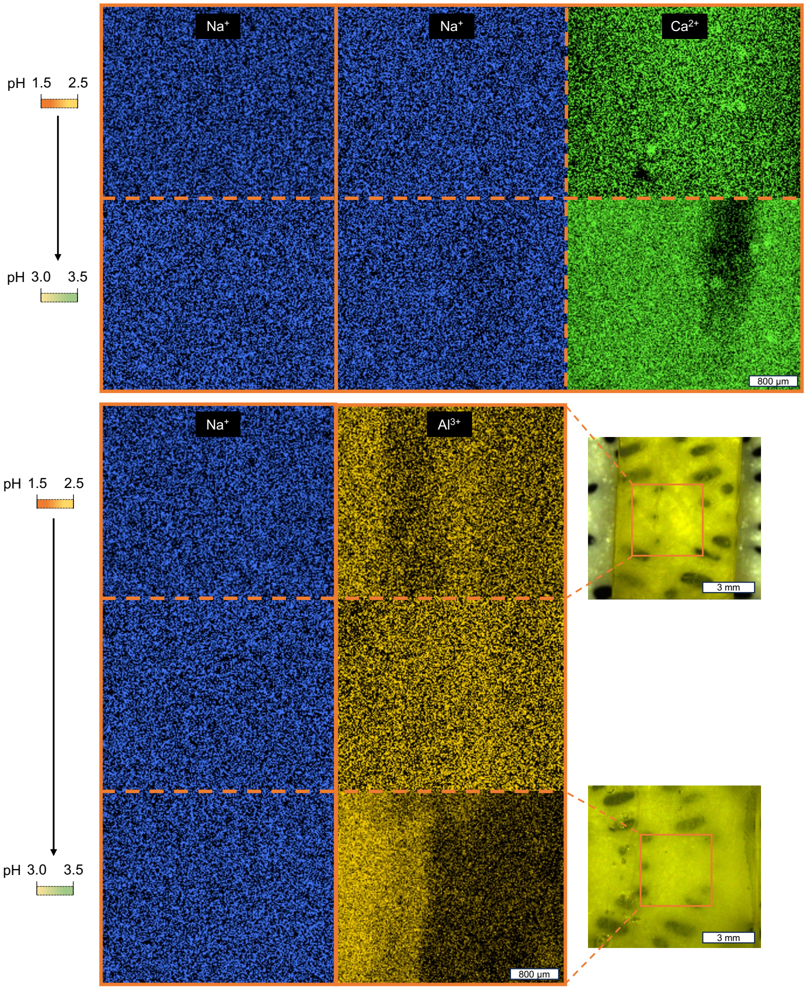

Supplementary Fig. 3: **X-Ray Fluorescence (XRF) mapping of additively manufactured MPEC.** Na^+ MPEC (top left, 1x2 pane) and Ca^{2+} MPEC (top right, 2x2 pane) and Al^{3+} (bottom, 2x3 pane) with sodium counter-ion from buffer. From top to bottom in each pane shows samples fabricated in most acidic to most alkaline conditions. pH changes from from 2.40 to 3.20 and 2.15 to 3.04 for Na^+ and Ca^{2+}, respectively, show largely uniform and homogeneous metal ion distributions. Similarly, in Al^{3+} samples at pH 2.55 or lower homogeneous distributions is observed, while at further increasing proton activity aluminum rich and aluminum poor regions become distinctly apparent. This behavior coincides with an observed contraction of spatial distribution of metal centers in good agreement with observed phase segregation seen in Al^{3+} MPECs density from pH 1.40 to 3.10. Outset shows large area map of XRF relative to total sample width. Spot size for all XRF maps is 20µm × 20µm.

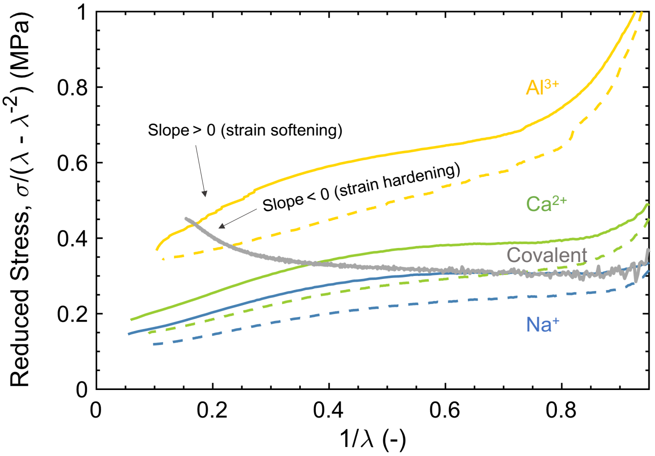

Supplementary Fig. 4: **Mooney-Rivlin representation of the stress-strain curves for uniaxial tensile experiments.** Reduced stress ($\sigma/(\lambda$ - λ^{-2})) versus inverse strain ($1/\lambda$) of uniaxial tensile response of MPEC with low pH (solid) and high pH (dashed). A positive slope of curves indicates the occurrence of strain-softening whereas a negative slope indicates the material undergoes strain-stiffening. Strain softening becomes more prominent for dynamically-crosslinked MPECs, indicating that physical bonds can dissociate along the applied deformation rate. For covalent network, due to permanent bonding, chain constraint release is not available, inducing strain hardening due to finite chain extensibility.

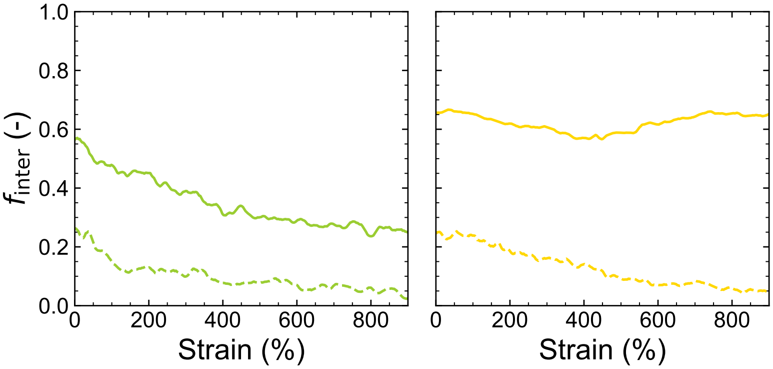

Supplementary Fig. 5: **Fraction of inter-crosslinked ions during deformation.** The change in fraction of inter-crosslinked divalent (left) and trivalent (right) ions during elastic deformations obtained using molecular dynamics simulations. Solid lines correspond to low pH regime and dashed lines correspond to high pH regime.

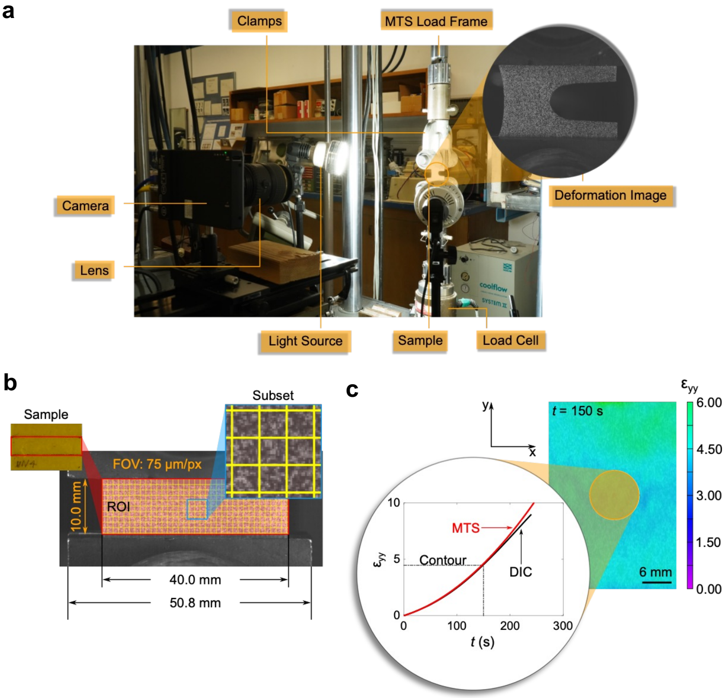

Supplementary Fig. 6: **Fracture set-up and analysis. a.** Experimental setup for tension testing using the MTS load frame. A series of flashlights are used to sufficiently and uniformly illuminate the sample for image capture using the high-resolution camera and DIC analysis. **b.** Overview of the DIC analysis with the region of interest (ROI), dimensions of the samples, cross-head width for calibration, and labeled subsets. **c.** Comparison of ε_{yy} calculated from actuator displacement data and averaged DIC data within the orange circle as a function of time for an un-notched sample undergoing uniaxial tension. Additionally, the strain field at $t = 150$s is shown to demonstrate the uniform distribution of ε_{yy} indicating no slip occurred.

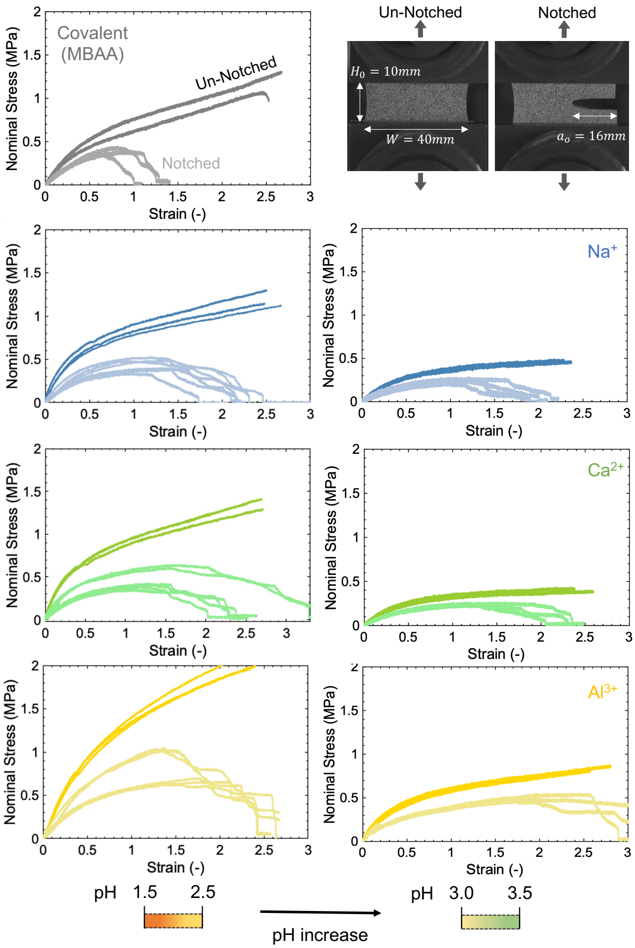

Supplementary Fig. 7: **Stress-strain curves for un-notched and notched MPEC for fracture experiment.** Un-notched and notched samples, with the dimensions of $W \times H_0 \times th$ to 40×10×1.5mm, were stretched uniaxially at a strain rate of $\dot{\varepsilon} \approx 0.02$ s^{-1}.

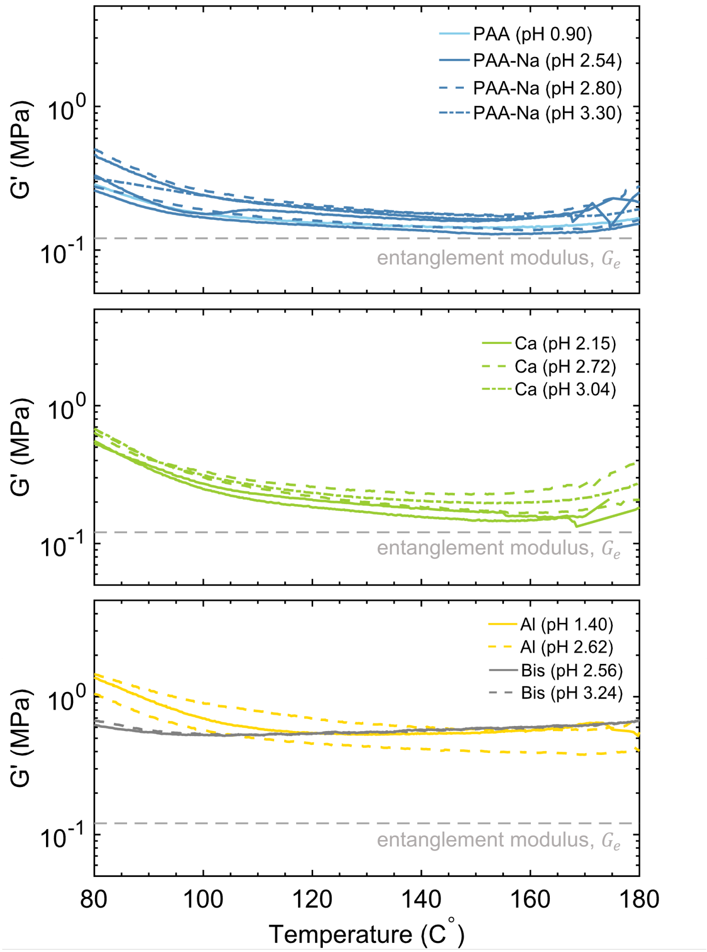

Supplementary Fig. 8: **Plateau modulus.** Storage modulus of MPECs measured in rubbery regime from 80°C to 180°C with the temperature ramp rate of 5°C/min. Due to the incomplete rubbery regime for water evaporation, an effective plateau modulus of MPECs was obtained from the storage modulus at $T_g \ll T \sim 150°C \leq T_{\text{exo-endo}}$ of the polymer in the rubbery state. Higher valency gels induced higher plateau modulus, deviating from the entanglement modulus limit (G_e) whereas different pH gels did not exhibit noticeable differences. G_e is analytically estimated [1]:$G_e = \frac{\rho RT}{M_e} \sim \frac{kT}{\nu_o N_e}$; $N_e \sim 100\frac{\nu_o^2}{b^6}$ with $\nu_o \approx$1.93 nm^3 and $b \approx 15.7$ Å [2]

.

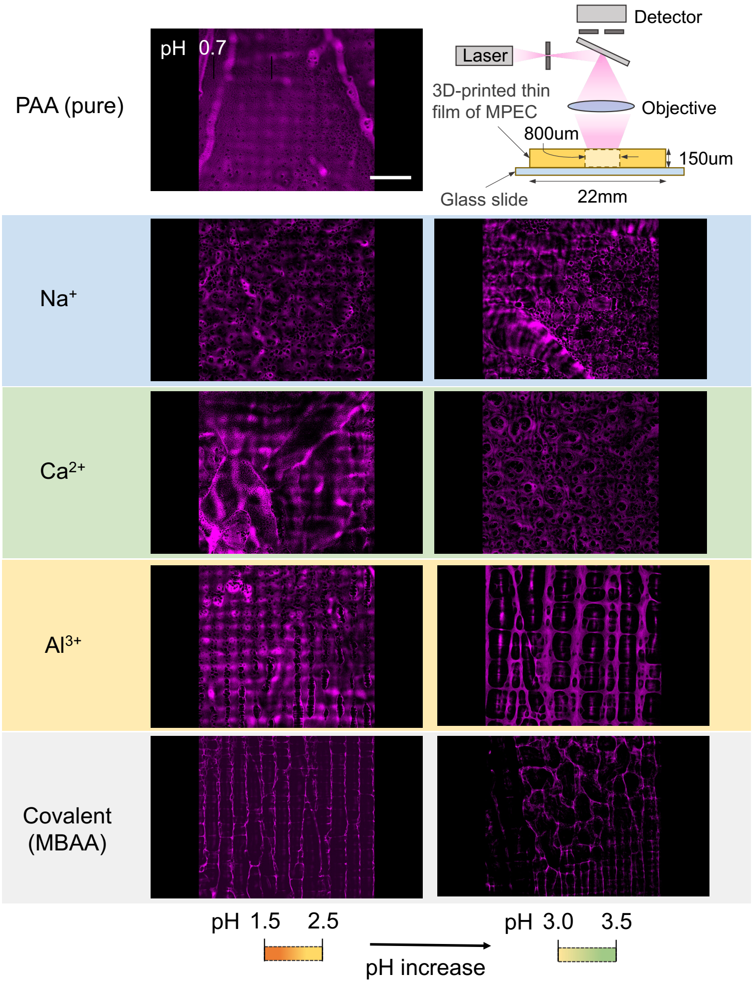

Supplementary Fig. 9: **Confocal Microscope Images of Additively Manufactured MPEC.** Confocal microscopy conducted at the depth of on pH-controlled MPEC samples showing Poly(Acrylic Acid) autofluorescence (purple) at 640 nm where negative void space corresponds to fluid filled regions. All samples are imaged at the depth of 50 $\pm$ 25 um of 3D-printed thin films of MPEC. Higher pH of the gels demonstrating severe phase separation of fluid filled regions from polymeric region for similar molecular weight network. Scale bar for all images 150um.

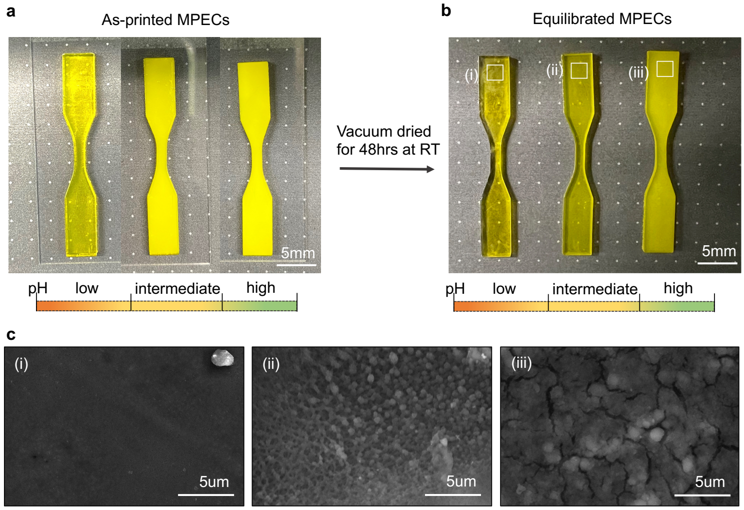

Supplementary Fig. 10: pH-dependent opacity and microstructure of additively manufactured MPEC. MPECs exhibit exhibit the features of phase separations of the dense polymeric network from the surrounding solvents or dilute phases as pH increases. The coexistence state of the gel at higher pH can be corroborated by the macroscopic opacity change of MPEC gels both in **a.** as-printed state and **b.** equilibrated state. **c.** Scanning Electron Microscope (SEM) micrographs capture the alteration of gel microstructure with the change in pH. Regardless of the cation types, all MPECs exhibit similar responses. Ca^{2+}-MPECs were shown as a representative.

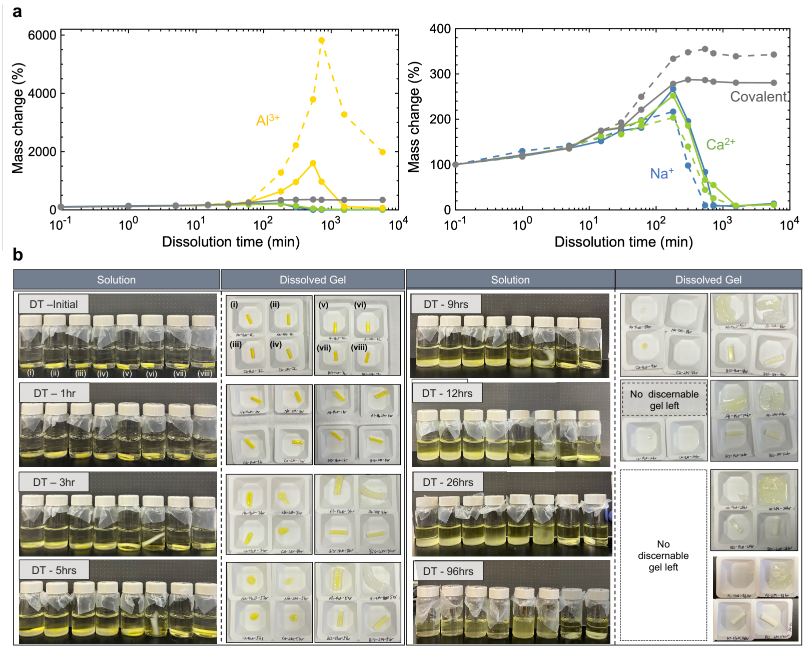

Supplementary Fig. 11: **MPEC swelling and dissolution progress.** 70mg of MPEC gels were dissolved in 10mL of miliQ H_2O (pH 7). **a.** The swelling and dissolution progress of MPECs at the low (solid) and intermediate pH (dashed) were monitored. Mass change of samples over the dissolution time were tracked by calculating $\Delta m\% = \frac{(m(t)-m_i)}{(m_i)} \times 100$ where m_i is the initial mass of equilibrated MPEC and $m(t)$ is the mass of current samples. For Al^{3+}-MPEC, we observed significant swelling before dissolution as slow relaxation of ions and polymers allows the gel to retain a shape yet the disentanglement of polymer clusters allows larger swelling degree. Then, once it reaches the maximum degree of swelling, the gel starts to dissolve. Conversely, as bond order of the associated metallo-polyelectrolyte complex drops below two, the dynamically crosslinked network begins to collapse and dissolve. As expected, the covalently crosslinked network initially swelled as the medium was introdued into the network and then reaches equilibrium swelling. The effects of charge sparsity was manifested by the faster dissolution and phase split for mono/divalent and trivalent gels, respectively. **b.** Optical images of MPECs when in solution and the remaining gels extracted from the supernatent liquid: (i),(ii)-Na^+-MPEC at low and intermediate pH (iii),(iv)-Ca^{2+}-MPEC at low and intermediate pH, (v),(vi)-Al^{3+}-MPEC at low and intermediate pH, and (vii),(viii)-MBAA(covalent)-MPEC at low and intermediate pH.

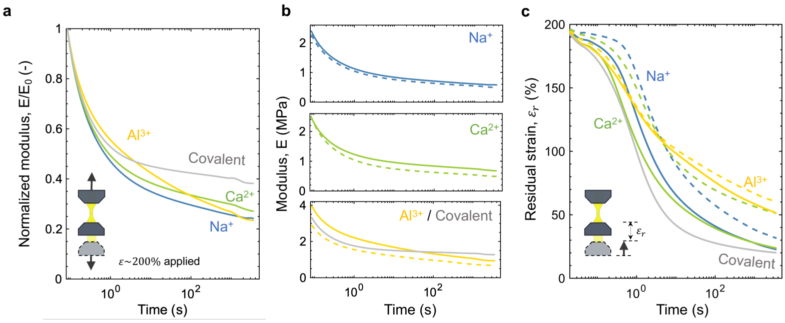

Supplementary Fig. 12: **Stress relaxation and recovery of MPECs.** Dog-bone shaped specimens with a length of 32mm, width of 1.6mm and gauge length of 4.765mm were used for stress relaxation and recovery experiments. **a,** Stress relaxation modulus and **b,** the normalized modulus, defined as the ratio of relaxation modulus (E) to the instantaneous modulus (E_0) of MPECs with low pH (solid line) and high pH (dashed line) were plotted over time. The samples were stretched under a strain of 200% in 0.1s and hold at the fixed strain for 1-hour at the ambient condition. Consistent with the MD simulations, Al^{3+}-MPECs exhibited slower relaxation compared to Na^+ and Ca^{2+}-MPECs. **c,** Residual strain (ε_r), was also tracked instantly after the release of the 1-hour hold at the fixed strain. Covalent gels exhibited the fastest and largest recovery whereas higher valency ions induced gels become more viscoplastic. Gel at higher pH (dashed line) also exhibit more viscoelastic/plastic behavior due to the segregated network from phase separation, compared to a single-phase gel at lower pH (solid line).

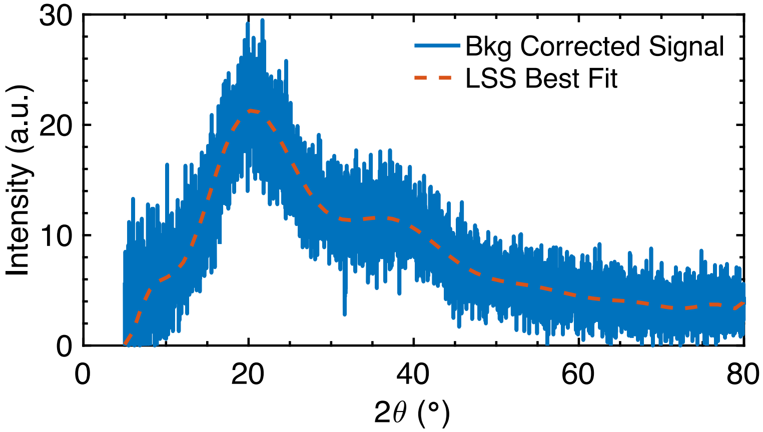

Supplementary Fig. 13: **Representative X-ray Diffraction (XRD) pattern of characteristic MPEC gel.** Small (10–15 mg) samples of MPEC gels were flash frozen using liquid nitrogen immediately upon reaching exothermic signature in DSC data. XRD for samples was run at a quick scan rate (3.5 $\frac{{}^\circ 2\theta}{\text{min}}$) from 2θ of 10–80° using a Rigaku SmartLab X-ray Diffractometer. Background scans were run under commensurate conditions immediately following sample run. Sample temperature was monitored using an laser infrared temperature probe to ensure samples remained below the onset of the glass transition region of the MPEC gels during the scan. All samples exhibited similar profiles, so that of Al^{3+}-MPEC is presented as representative. MPEC gels exhibit traditional peaks associated with disordered carbon content (polymer backbone) and water content with no obviously apparent crystallization being observed in the samples. Accordingly, the exothermic event observed in DSC is not believed to correspond to polymer crystallization.

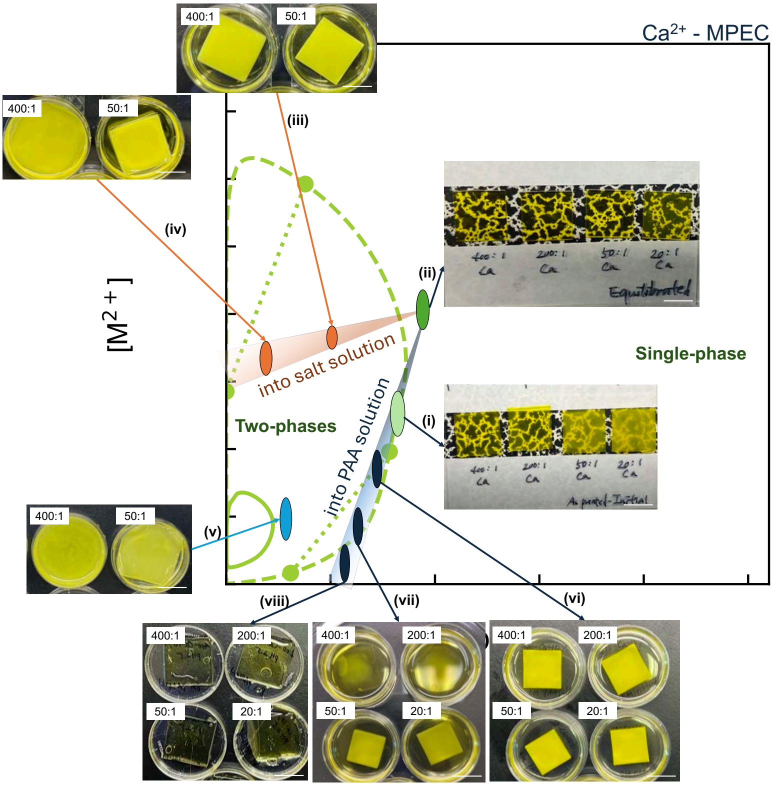

Supplementary Fig. 14: **Experimental validation of the phase diagram for Ca-MPEC.** To explore different phase regions on the diagram, equilibrated Ca-gels with different density (400:1, 200:1, 50:1, 20:1 for AA:Metal ratio) were immersed in various solutions, including water, calcium nitrate solutions with different concentrations, and polyacrylic acid solutions with different concentrations, until equilibrium was reached. Different phases were observed depending on the solution type and concentration. aligning with the behavior predicted from the phase diagram. Gels become opaque in two-phase region whereas remain transparent in the single-phase region. (i) As-printed gels; (ii) Equilibrated gels after post-processing; (iii) and (iv) immersed in 3ml and 6ml of 1.5M Ca(NO3)2 solution; (v) immersed in water; (vi), (vii) and (viii) immersed in 3ml, 6ml, 8ml of 8M PAA solution (M_w~5kDa). Scale bar for all images 10mm.

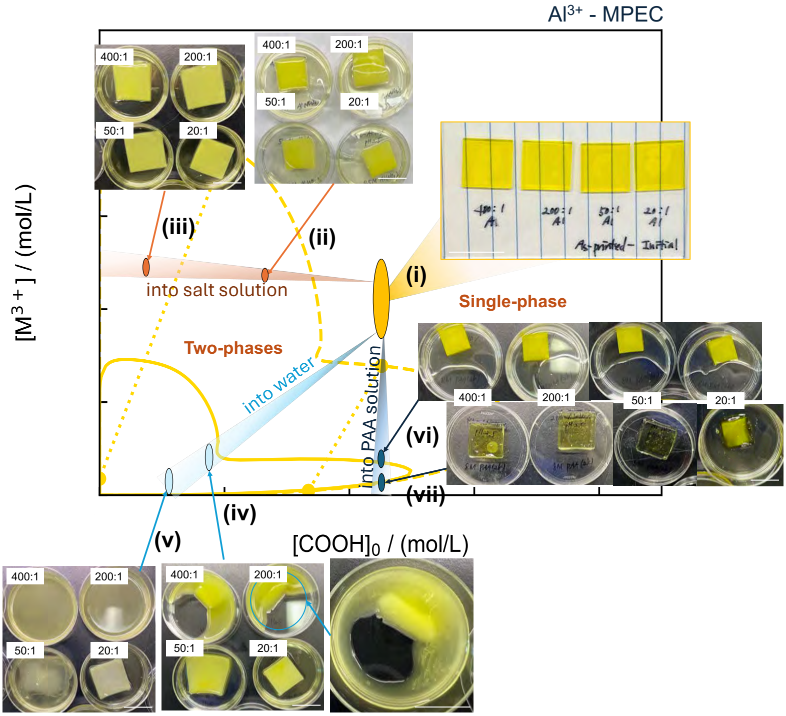

Supplementary Fig. 15: **Experimental validation of the phase diagram for Al-MPEC.** To explore different phase regions on the diagram, equilibrated Al-gels with different density (400:1, 200:1, 50:1, 20:1 for AA:Metal ratio) were immersed in various solutions, including water, calcium nitrate solutions with different concentrations, and polyacrylic acid solutions with different concentrations, until equilibrium was reached. Different phases were observed depending on the solution type and concentration. aligning with the behavior predicted from the phase diagram. Gels become opaque in two-phase region whereas remain transparent in the single-phase region. (i) Equilibrated gels after post-processing; (ii) and (iii) immersed in 3ml and 6ml of 1M Al(NO3)3 solution; (iv) and (v) immersed in 4ml and 8ml of water; (vi) and (vii) immersed in 3ml, 6ml of 8M PAA solution (M_w~5kDa). Scale bar for all images 10mm.

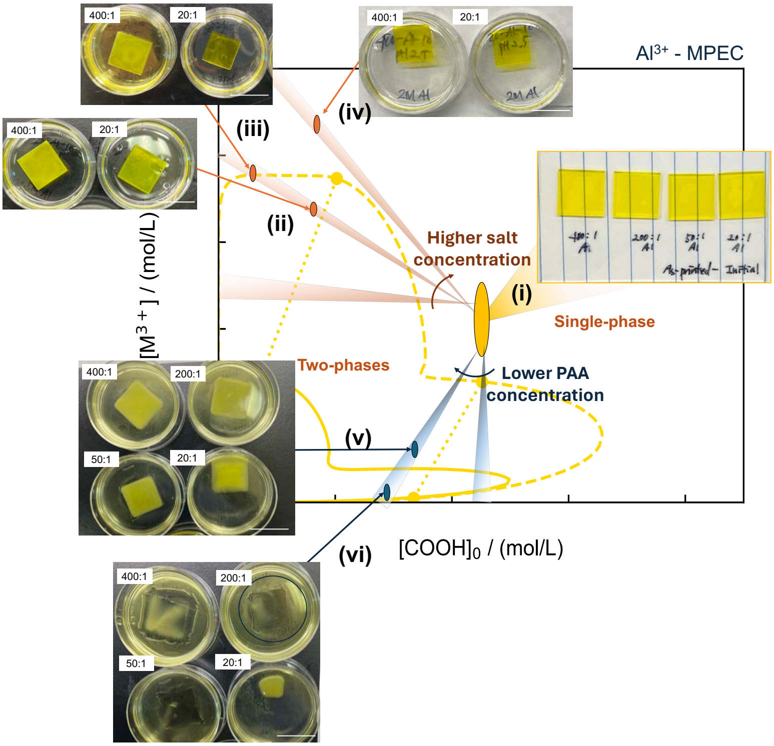

Supplementary Fig. 16: **Additional experimental validation of the constructed phase diagram for Al-MPEC.** (i) Equilibrated gels after post-processing; (ii), (iii), (iv) immersed in 3ml of 1.5M, 1.75M and 2M Al(NO3)3 solution; (v) and (vi) immersed in 4ml and 8ml of 4M PAA solution (M_w~5kDa). Scale bar for all images 10mm.

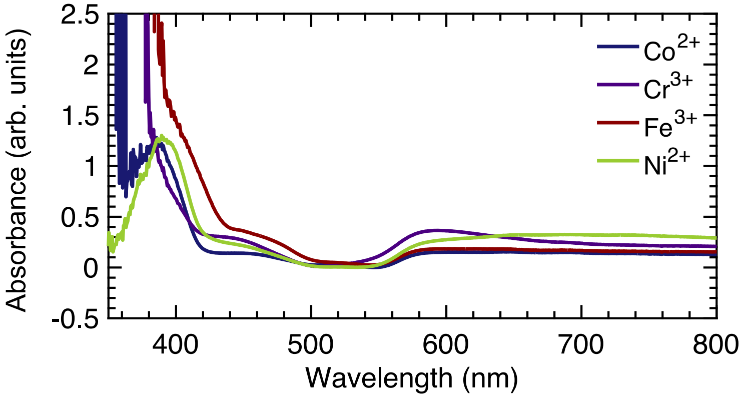

Supplementary Fig. 17: UV-Visible photospectroscopy of relevant, transition metals with color arising from d-d orbital electronic interactions and ligand-to-metal charge transfer of solid-state MPEC gels in transmission mode. The color of gel samples (e.g. Cobalt II, Nickel II, Chromium III, Iron III) supports an octahedral (O_h) coordination environment of their ligand fields, favoring bidentate chelation in the case of symmetric O_h field.

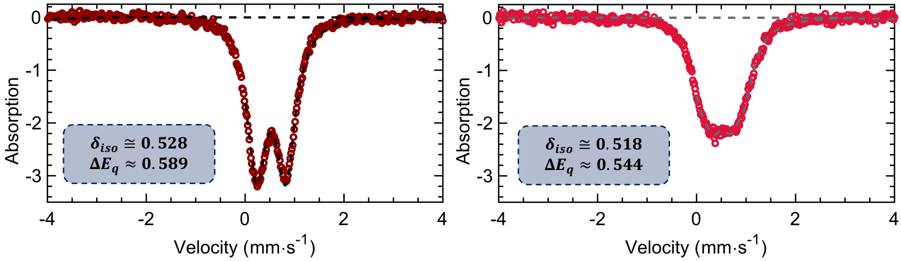

Supplementary Fig. 18: ^{57}Co-Moessbauer spectrometry of Fe(III) gel phase and frozen resin (solution) phase measure an isomer shift of $\delta_{iso} \approx 0.528$ and $\delta_{iso} \approx 0.518$ with a quadrupole splitting of $\Delta E_Q \approx 0.589$ and $\Delta E_Q \approx 0.544$, respectively, consistent with symmetric, octahedral coordination of the Fe^{3+} center[3–6]. Given the common ionic substitutional nature of Fe(III) with other trivalent species, in particular Al(III) with its coordination modes to PAA measured by FTIR in this study, we believe that both trivalent species are consistently bidentate chelated by carboxylates.

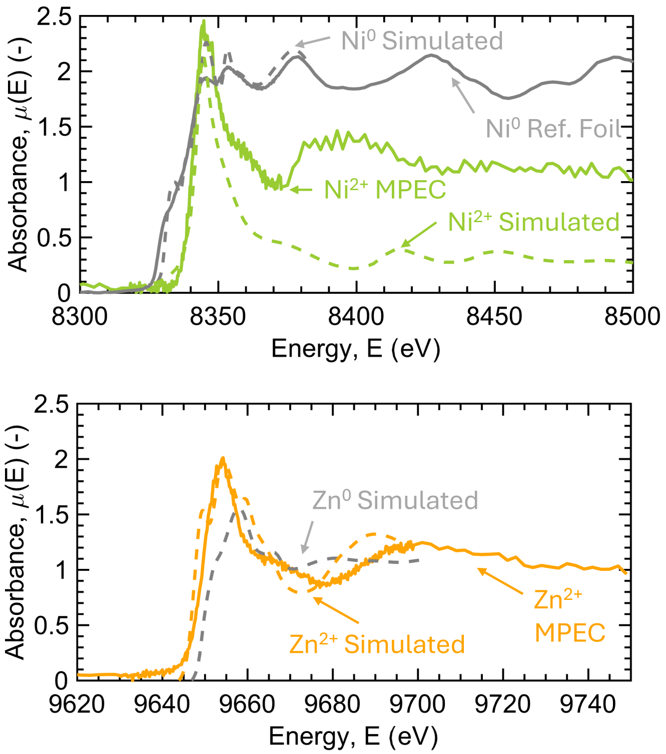

Supplementary Fig. 19: Near edge X-ray absorption (XANES) spectra of Zn^{2+} and Ni^{2+} in a preliminary study of coordinating environment using EasyXAFS300+ overlaid with FFEF *ab initio* multi-scattering simulations. Extended spectra were insufficiently resolved to interpret in-detail, but the near edge spectrum is indicative of octahedral coordination environments of appropriate valence species in all MPEC gels the EasyXAFS instrument was capable of measuring.

Supplementary Tables

**Reagent**	**Purpose**	**Amount**
Acrylic acid	Monomer	15 mL
2M Sodium acrylate	Monomer/Buffer	2.18 mL
H_2O / 2M NaOH / 4M NaOH	Diluent/Buffer	2.40 mL
Nitrate Salt (6M for Mono, 3M for Di, 2M for Trivalent)	Binder	0.73 mL
N,N-dimethylformamide (DMF)	Diluent	1.37 mL
2,4,6-Trimethylbenzoyldi-Phenylphosphinate (TPO-L)	Photoinitiator	695.3 mg
Trisodium 5-hydroxy-1-(4-sulfonatophenyl)-4-[(E)-(4-sulfonatophenyl)diazenyl]-1H-pyrazole-3-carboxylate (Tartrazine)	UV blocker	8.15 mg
Glycerol	Plasticizer	2.40 mL

Supplementary Table 1: MPEC photoresin composition[1].

[1] for 25ml of total resin volume

Parameter	Setting
UV wavelength	405 nm
Layer thickness	50 µm
Exposure time	30s
Built head lifting speed	60 mm/min
Drying conditions	24hrs at 25°C, Vacuum
Ambient equilibration conditions	48-72hrs[2] at RT, 45% RH

Supplementary Table 2: LCD printing and post-processing parameters.

Metal ions	Diluent/Buffer[3]	pH[4]
Na^{+}	H_2O	2.55
	2M NaOH	2.95
	4M NaOH	3.25
Ca^{2+}	H_2O	2.15
	2M NaOH	2.72
	4M NaOH	3.10
Al^{3+}	H_2O	1.40
	2M NaOH	2.40
	4M NaOH	2.97
Ni^{2+}	H_2O	2.06
	2M NaOH	2.62
	4M NaOH	3.05
Co^{2+}	H_2O	2.15
	4M NaOH	2.95
Fe^{3+}	H_2O	0.70
	4M NaOH	2.84
Cr^{3+}	H_2O	1.55
	4M NaOH	3.00

Supplementary Table 3: pH measurement of photoresin.

[2] High hydrophilicity of Polyacrylic acid results in high sensitivity to temperature and RH. Thus, equilibration conditions were slightly varied depending on the temperature and RH conditions.

[3] to keep the constant concentrations of all chemical species in the photoresin, the same volume of diluent/buffer was added as indicated on Supplementary Table 1.

[4] pH was measured without the photoinitiator and glycerol to avoid the reading errors induced by the organic solvents.

**Gel**	**pH**	**Phosphorus**	**Na^+**	**[M^n+]**
PAA	0.9	7.6323E+04	2.7100E+02	
	2	3.6869E+04	7.7300E+02	
	2.54	7.1893E+04	8.6400E+02	
	2.8	5.7893E+04	1.2630E+03	
	3.25	4.5382E+04	1.6520E+03	
MBAA	0.9	2.0239E+05	5.8330E+03	
Na^+	2.4	7.7519E+05	6.2780E+03	
	3.2	1.4698E+06	6.2425E+04	
Ca^{2+}	2.15	8.0942E+04	1.8540E+03	4.7704E+05
	3.04	2.6679E+06	4.5130E+04	7.2856E+06
Al^{3+}	1.4	4.8188E+05	3.9330E+03	6.1579E+04
	2.55	1.3306E+06	3.4479E+04	4.3611E+05
	3.1	9.2093E+05	4.9520E+04	4.1606E+05

Supplementary Table 4: **XRF Counts in constant volume mapping volume across cation and pH ranges tested.** Counts are reported for identical area (3.5 mm $\times$ 3.5 mm), sample thickness (2.5 mm), resolution (20 μm), and dwell time (40 ms/px) under 50kV, 600 μA conditions.

Supplementary Discussions

1 Binding Energy of Metal-Acetate Complexes

For the majority of our work, we have comfortably assumed that the binding between metal ions and charged sites is mediated purely by Coulombic interactions, which would scale only with the metal ion valency and radius. While there is no way to test this assumption using the coarse-grained methods used previously, one can leverage other tools to validate this assumption. In this case, we choose to use quantum chemistry methods. Specifically, quantum density functional theory (qDFT) where, based on the benchmarks performed by Blasko et al. [7], we have decided to use the B3LYP[8] qDFT functional with the D3(BJ) dispersion correction [9] and def2-TZVPP basis set [10, 11], as provided by the ORCA package [7]. Further details regarding the qDFT simulations have been provided within the Methods.

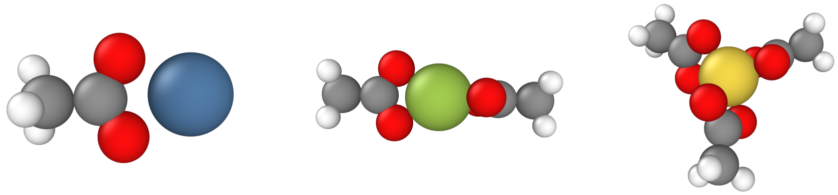

(a) Monovalent system (b) Divalent system (c) Trivalent system

Supplementary Fig. 20: Initial configurations of **a,** monvalent, **b,** divalent, and **c,** trivalent system, used in all quantum chemistry calculations to obtain binding energies between acetate and metal ions.

1.1 Binding energy

In the first instance, we consider obtaining the binding energy between acetate monomers (as poly(acetate) is the primary system of interest) and various metal ions. Although this is likely to be a poor approximation, it should be sufficient to provide an initial qualitative understanding of the interaction between charged sites and metal ions. To best reflect the favored configuration of each metal ion valency, we ensure that the number of acetates in the system is sufficient to neutralise the metal ion, as shown in Supplementary Fig. 20. As a first-order approximation, the solvent, in this case water, is approximated using the Conductor-like Polarizable Continuum Model (CPCM) [12].

To then measure the actual binding energy, we first optimise the structure of the complexes shown in Supplementary Fig. 20 and obtain the final single point energy (E_{complex}). We then compute the single point energy of the ion by itself (E_m). Finally, we use the optimised configuration determined previously,

remove the metal ion from the system, re-optimise the configuration of the remaining acetates and obtain the final single point energy of this system (E_{acetate}). The binding energy, ΔE_{bind}, is then obtained as:

$$\Delta E_{\text{bind}} = E_{\text{complex}} - E_m - E_{\text{acetate}} \, . \tag{S.1}$$

This process is illustrated visually in Supplementary Fig. 21. To determine the contact separation, the average center-of-mass distance between acetates and metal ions was used.

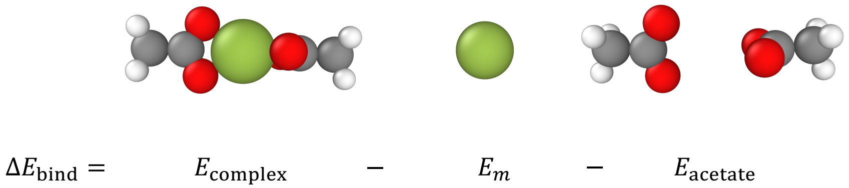

Supplementary Fig. 21: Visual representation of the process used to obtain binding energies from quantum chemistry calculations in the case of a divalent cation.

Using this process, we are able to obtain the binding energies shown in Supplementary Fig. 22. To validate these results, we repeat these calculations using the ωB97X-D3 [13, 14] functional in the case of sodium, calcium and aluminium ions. As we can see, the results obtained for both functionals are in good agreement.

These binding energies generally match our expectations, where trivalent ions have the strongest binding energy and monovalent ions have the weakest. If the binding energy is normalised by the metal ion valency, we can see in Supplementary Fig. 22b that the binding energies collapse onto a single curve. Furthermore, generally, we observe a monotonic decrease in binding energy with metal-acetate separation. However, the scaling power of the binding energy with this distance is not -1. The scaling power is closer to -2, indicative of a ion-dipole interaction. In fact, if one were to use to dipole of acetate obtained from the optimisation configurations, as shown by the dashed lines in Supplementary Fig. 22, the predictions line-up exceptionally well with the calculated binding energies. This is slightly concerning for our assumption of the Coulombic potential governing binding energy in these systems.

Nevertheless, the key conclusion from this section is, in terms of the quantitative impact of the metal ion on the binding energy, this can be captured using solely the metal ion valency and radius.

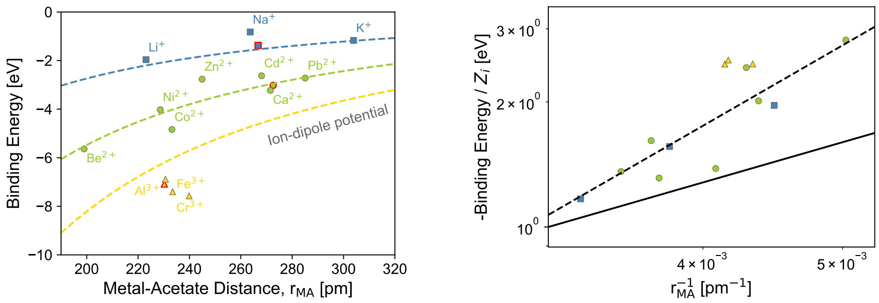

(a) (b)

Supplementary Fig. 22: **a,** Binding energies of different metal species obtained from quantum chemistry calculations. Symbols with a grey edge were calculated using the B3LYP-D3(BJ) functional and symbols with a red edge were calculated using the ωB97X-D3 functional. **b,** Normalized binding energies with the corresponding metal ion valency collapse onto a single curve. Dashed lines represent the predictions from the ion-dipole potential and the solid line represents predictions from the Coulombic potential. The color scheme used in this figure follow the scheme used in the main text (blue: monovalent, green: divalent and yellow: trivalent ions).

1.2 Molecular orbitals

A noteworthy observation made during the optimisation of the metal complexes was that, to converge the nickel, cobalt, iron and chromium calculations, the spin of the system between the lone ion and the ion within the complex had to be reduced, indicating that the metal ions may now be sharing electrons from their half-filled orbitals with the acetates, forming a shared molecular orbital. Indeed, if we first consider the divalent ions, when comparing the optimised complex structures for nickel and zinc in Supplementary Fig. 23, we can see that, despite starting with the carboxylate functional groups perpendicular to one another, in the case of the nickel ion, the preferred configuration is when these functional groups are parallel. This is rather strange as this configuration does not minimise the electrostatic repulsions between the electronegative oxygen atoms. However, Supplementary Fig. 23c illuminates why this configuration is preferred: in this configuration, and likely due to nickel's half-filled orbitals, the complex is able to form a delocalised orbital that encompasses the two oxygen atoms and central metal ion. This orbital is most-likely compensating for the electrostatic repulsions between the oxygens. The orbital is possibly also responsible for the slightly stronger binding energy in contrast to divalent metal ions with filled orbitals.

However, in the case of the trivalent ions, we can see that there isn't as significant a change between the metals with half-filled orbitals and those with filled orbitals. Furthermore, when optimising the geometries of the complexes,

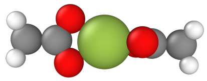

(a) Zinc

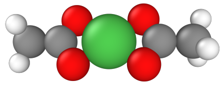

(b) Nickel

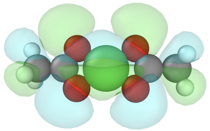

(c) Nickel Molecular Orbital

Supplementary Fig. 23: Optimised complexes for **a,** zinc and **b,** nickel and **c,** Nickel molecular orbitals obtain from quantum chemistry calculations.

the final configurations for all cases was not too dissimilar to the initial structure shown in Supplementary Fig. 20c. This is most likely because, even in the case where the spin is reduced between the lone metal and metal complex, the repulsions between oxygen atoms is too great. As a result, the optimal configuration is always the one that minimises these repulsions. The small differences in binding energy are most likely still a result of sharing of electrons between the metal ions and the acetates.

2 Impact of metal ion identity

While the main body of the manuscript focused solely on the Na^+, Ca^{2+} and Al^{3+} ions to represent the impact of the dynamic crosslinker valency, the fabrication process proposed in the work is not limited to these ions. Indeed, we have experimentally and theoretically explored the impact of different ions. These results are presented here.

2.1 Theoretical predictions

Following the results from Supplementary Fig. 22a, we expect the metal ion identity to be captured by two factors: valency and ion diameter. The effect of ion valency has already been discussed in-depth within the main text. However, by repeating the molecular dynamics simulations performed in the main text with different ion diameters for the metal crosslinker, the results in Supplementary Figure 24 are obtained. To cover the maximum effect that could arise from varying the ion diameter, two diameters were used: 0.5σ and σ. In real units, this would be the equivalent of changing the metal ion from Beryllium to Lead, for the divalent ions.

As shown in supplementary figure 24a, by increasing the ion diameter, the mechanical stiffness of the gels has been reduced. To understand the reason for this, consider supplementary figures 24b-24d, where the increased ion diameter resulted in faster relaxation times for both the ion-pair and end-to-end vector autocorrelation functions, arising from the weaker binding. This highlights that the polymer chains are now more mobile, allowing them to re-arrange faster under deformation, thus experiencing less stress.

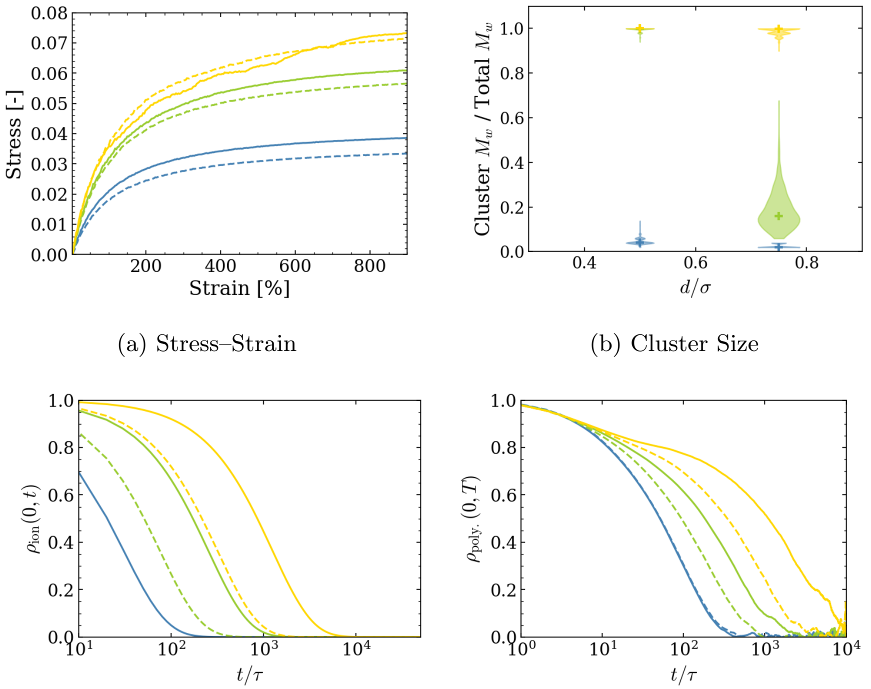

(a) Stress–Strain

(b) Cluster Size

(c) Ion-pair relaxation

(d) End-to-end vector relaxation

Supplementary Fig. 24: Results obtained from molecular dynamics simulations of MPEC gels with varying ion diameters: **a,** Stress-strain curves from uniaxial stretching. **b,** Cluster size distribution over different ion diameters. **c,** Ion-pair relaxation and **d,** end-to-end vector relaxation. Solid curves correspond to diameters of 0.5σ and dashed curves correspond to diameters of σ. The color scheme used in this figure follow the scheme used in the main text (blue: monovalent, green: divalent and yellow: trivalent ions).

There are some additional observations to be made. Interestingly, the monovalent end-to-end autocorrelation function is almost unaffected by the change in ion diameter. This can be rationalized by the fact that, in the monovalent gels, as they cannot crosslink different chains, the ion pairs are not contributing to the gel network formation. As such, a change in the binding strength will not affect the polymer motion. The differences in stress-strain curves arises from additional repulsion between chains as, whatever counterion condensation had been occuring before has now been reduced, leading to a weaker response.

Another observation is that, despite the change in ion diameter, the trivalent stress-strain curve is not significantly affected. Although the relaxations times for the ion-pair and end-to-end vectors are decreasing, as observed in

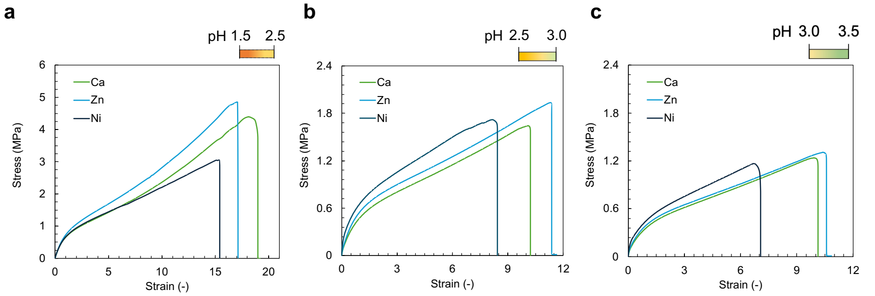

Sample	Modulus (MPa)	Std. Modulus (MPa)
Ca	1.259	0.129
Zn	1.191	0.065
Ni	1.395	0.303

Sample	Modulus (MPa)	Std. Modulus (MPa)
Ca	1.183	0.119
Zn	1.317	0.031
Ni	2.359	0.363

Sample	Modulus (MPa)	Std. Modulus (MPa)
Ca	1.155	0.191
Zn	1.329	0.066
Ni	2.110	0.319

Supplementary Fig. 25: Stress-strain curves obtained for different divalent metal ions (Ca^{2+}, Zn^{2+} and Ni^{2+}) at varying pH (**a,**: low, **b,**: medium, **c,**: high). The respective young's moduli and standard deviations of each gel have been given in the tables above. The same protocol was used to print and perform the experiments on the gels as described in the main text.

supplementary figure 24b, the cluster formation in the case of the trivalent gels is not as significantly affected as the divalent ions. This is most likely because of the relaxation times are still slower than those of the base divalent gel, and the fact that the trivalent ions can still form three crosslinks.

2.2 Experimental observations

To experimentally observe the impact of the choice of metal ion, we printed gels using different metal ions (from the same starting pre-gel solutions), confirmed their coordination geometry and implied chelation by carboxylate, and subsequently performed quasi-static tensile tests to measure their mechanical responses. The results are summarized in figures 25 and 26. Spectroscopic interrogation confirming coordination of some characteristic species is documented in figures 17, 18, and 19.

Initially considering the divalent ions in figure 25, we indeed observe that the gels become stiffer when the ionic radii of the metal ion is reduced from Ca^{2+} to Zn^{2+} to Ni^{2+} at medium (figure 25b) to high pH (figure 25c), aligning with the theoretic predictions made in the previous section. Ni^{2+} demonstrates a significant increase in stiffness compared to Zn^{2+}, likely because, as discussed in Section 1.2, Ni^{2+} has different binding orbitals due to its half-filled d-orbitals. This distinct binding characteristic contributes to enhanced binding strength and relaxation. Importantly, this effect is only manifest in the binding strength differences as UV-Visible absorption and near-edge X-ray absorption spectra appear consistent with a symmetric (on-average) coordination sphere by carboxylate and water (weak field ligands), as shown in figures 17 and 19.

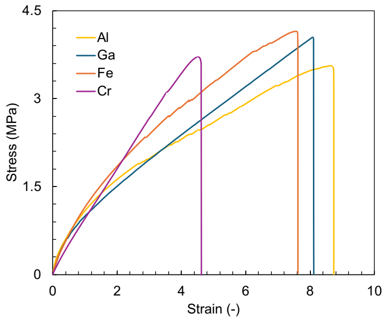

Sample	Modulus (MPa)
Al	2.817
Ga	1.984
Fe	2.026
Cr	1.293

Supplementary Fig. 26: Stress-strain curves obtained for different trivalent metal ions (Al^{3+}, Ga^{3+}, Fe^{3+} and Cr^{3+}). The respective young's moduli and standard deviations of each gel have been given in the table on the right. The same protocol was used to print and perform the experiments on the gels as described in the main text.

Thus, we attribute observed changes to bond character and strength rather than these effects in concert with coordination environment changes.

Interestingly, there appears to be a maximum in the tensile critical strain as we vary the ionic radius, likely resulting from a balance between the binding strength of the coordinating bonds and the polymer relaxation time. An interesting exception to the stiffness trend occurs at low pH, where the trend does not strictly follow ion size. This suggests that protonation and other charge interactions become more dominant, and the difference in binding strength becomes less detectable, especially in the low pH regime where the number of available binding sites is reduced and protonation becomes strongly favored.

For the trivalent ions shown in figure 26, as predicted in figure 22, the binding strength of all trivalent ions is quite similar, resulting in comparable mechanical responses among the different trivalent ions. However, Cr^{3+}-MPEC gels, while initially showing a very similar mechanical response, fail earlier than the other trivalent gels. As seen in **Fig. 1b** of the main text, Cr^{3+}-MPEC gels can be printed with much finer resolution than the other gels. This likely arises from the fact that Cr^{3+} has multiple half-filled d-orbitals, leading to a binding characteristic closer to covalent than ionic. These stronger bonds result in a more static nature in the crosslinking, causing strain stiffening and earlier failure due to altered cluster formation and distribution. Again, in the case of Cr^{3+}, the coordination environment appears consistent between ions, with color indicating an averagely symmetric octahedral environment coordinated by carboxylates and/or water.

Interestingly, Fe^{3+} also has multiple half-filled d-orbitals but becomes less stiff compared to hard ions such as Al^{3+} or Ga^{3+}, failing at approximately the same strain as the other trivalent gels. While there is some suggestion in literature regarding photo-reduction of ferric iron to ferrous iron in the presence of a supporting electrolyte (Na^{+}) and carboxylic acids, hydroxycarboxylic

acids, and similar weak-field ligand environments[15–17], we see neither an indication of the presence of Fe^{2+} in resin or gel photo-spectroscopic data in UV-Visible transmission nor any intervalence charge charge transfer bands. To fully understand the impact of any local asymmetry in complex coordination environments on gel behavior or mixed ligand-fields with partial hydrated coordination (i.e. mixed carboxylate, carboxyl, and aqua ligand coordination of the cation), a more sophisticated computational model is needed to accurately capture their intricate interactions. As was the case for other metal species, Fe^{3+} gels show a symmetric octahedral coordinating environment in UV-Visible spectroscopy 17. Further, we see similar isomer shift and quadrupole splitting between solution and gel states of ferric iron samples in ^{57}Co-Moessbauer spectra, as shown in figure 18, agreeing well with a symmetric, octahedral coordination of the metal cation.

3 Modified Henderson–Hasselbach Equation

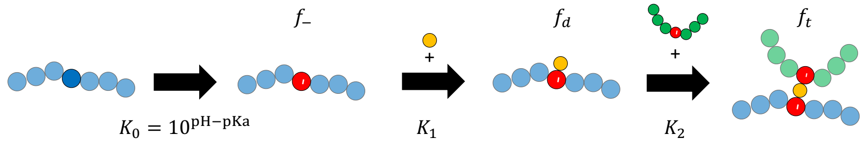

Supplementary Fig. 27: Visual representation of the possible associations of a weak polyelectrolyte in the presence of multivalent cations. Association constants are denoted by K_i and the fraction of polymers existing in state i are denoted by f_i.

If we treat our deprotonation equilibrium as a simple acid-base reaction, then the ratio of deprotonated (f_-) and protonated ($1 - f_-$) PAA sites can be obtained using the Henderson–Hasselbach equation:

$$\text{pH-pKa} = -\log\left(\frac{f_-}{1 - f_-}\right) . \tag{S.2}$$

While this equation has origins within solution chemistry, it can also be derived using statistical mechanics. Starting from a chain with m_p beads, we assume that m_- have been deprotonated. As such, the partition function for this system is given by:

$$Z_- = Z_{\text{ref.}} P_{-,\text{comb.}} K_0^{m_-} , \tag{S.3}$$

where $Z_{\text{ref.}}$ is our reference partition function. The combinatorial term, $P_{-,\text{comb.}}$ is given by:

$$P_{-,\text{comb.}} = \frac{m_p!}{(m_p - m_-)! m_-!} , \tag{S.4}$$

and the equilibrium constant, K_0, is simply given by:

$$K_0 = \rho_p 10^{(\text{pH-pKa})} \,. \tag{S.5}$$

If we were to minimise equation S.3 with respect to m_- and define a new variable, $f_- = m_-/m_p$, we would recover the Henderson–Hasselbach equation:

$$\text{pH-pKa} = -\log\left(\frac{f_-}{1-f_-}\right) \,. \tag{S.6}$$

However, if we want to modify this equation accounting for the fact that the deprotonated sites can subsequently bind to metal ions (and form crosslinks), we need to modify our partition function. We now assume that, from m_m metal cations and of the m_- charged sites on the polyelectrolyte backbone, m_d have formed dimers. Our partition function, now starting from Z_- as the reference system, is given by:

$$Z_d = Z_- P_{d,\text{comb.}} K_1^{m_d} \,, \tag{S.7}$$

where the combinatorial term is given by:

$$P_{d,\text{comb.}} = \frac{m_-!}{(m_- - m_d)!m_d!} \frac{m_m!}{(m_m - m_d)!m_d!} m_d! \,. \tag{S.8}$$

The additional $m_d!$ contribution arises from the fact that there are $m_d!$ ways to select the dimers. The equilibrium constant for this association, following equation S.44, is given by:

$$K_1 = \rho_p \kappa_d \sigma_{pm}^3 g^{\text{EXP}}(\sigma_{pm}) \left[\exp\left(\frac{l_\text{B} Z_m}{\sigma_{pm}}\right) - 1\right] \,, \tag{S.9}$$

where κ_d is the bonding volume for this association. We can repeat the above process to account for the formation of m_t trimers from m_d dimers and $m_- - m_d$ unassociated charged sites, and even for higher-order 4-mers. Saving some unnecessary algebra, the resulting expression, starting from the original reference partition function $Z_{\text{ref.}}$, is:

$$\begin{aligned} Z_q = {} & Z_{\text{ref.}} K_0^{m_-} K_1^{m_d} K_2^{m_t} K_3^{m_q} \\ & \times \frac{m_p! m_m!}{(m_p - m_-)!(m_m - m_d)!(m_t - m_q)!(m_- - m_d - m_t - m_q)!m_q!} \,, \end{aligned} \tag{S.10}$$

where:

$$K_2 = \rho_p \kappa_t \sigma_{pm}^3 g^{\text{EXP}}(\sigma_{pm}) \left[\exp\left(\frac{l_\text{B}}{\sigma_{pm}}\left[Z_m - \frac{1}{2}\right]\right) - 1\right] \,, \tag{S.11}$$

$$K_3 = \rho_p \kappa_q \sigma_{pm}^3 g^{\text{EXP}}(\sigma_{pm}) \left[\exp\left(\frac{l_\text{B}}{\sigma_{pm}}\left[Z_m - \frac{2}{\sqrt{3}}\right]\right) - 1\right] \,, \tag{S.12}$$

where κ_t and κ_q are the bonding volume for each association. Note that, with each incremental association, the binding energy is reduced due to the local repulsions from the sites that are already associated. The magnitude of these reductions was determined by the configurations which resulted in the least repulsion between charged sites (linear for the trimer and triangular planar for the 4-mer). This will lead to a situation where $K_3 < K_2 < K_1$, which should make sense as, when forming additional associations, both the local repulsion and steric hindrance will make these interactions less favorable.

If we now minimise the above expression with respect to m_-, m_d, m_t and m_q, and normalise these variables with respect to the number of beads ($f_i = m_i/m_p$), we obtain the following:

$$K_0 = \frac{f_- - f_d - f_t - f_q}{1 - f_-}\,, \tag{S.13}$$

$$K_1 = \frac{f_d - f_t}{(f_m - f_d)(f_- - f_d - f_t - f_q)}\,, \tag{S.14}$$

$$K_2 = \frac{f_t - f_q}{(f_d - f_t)(f_- - f_d - f_t - f_q)}\,,$$

$$K_3 = \frac{f_q}{(f_t - f_q)(f_- - f_d - f_t - f_q)}\,.$$

Unfortunately, the variables f_i are not particularly useful as, for example, f_d measures the fraction of sites that have at least formed a dimer, not only. As such, we define the following fractions, X_i, which measure the fraction of sites in state i only:

$$X_- = f_- - f_d - f_t - f_q\,, \tag{S.15}$$

$$X_d = f_d - f_t - f_q\,, \tag{S.16}$$

$$X_t = 2(f_t - f_q)\,,$$

$$X_q = 3f_q\,.$$

We can also define the fraction of metal ions not associated as:

$$X_{m,\text{free}} = f_m - X_d - X_t/2 - X_q/3\,. \tag{S.17}$$

Substituting into equations S.13 gives:

$$K_0 = \frac{X_-}{1 - X_- - X_d - X_t - X_q}\,, \tag{S.18}$$

$$K_1 = \frac{X_d}{X_{m,\text{free}} X_-}\,, \tag{S.19}$$

$$2K_2 = \frac{X_t}{X_d X_-}\,,$$

$$\frac{3}{2}K_3 = \frac{X_q}{X_t X_-}\,.$$

As we can see in the above system of equations, we are representing the set of associations in Supplementary Fig. 27 as sequential, reversible reactions. Indeed, without even implementing the above system of equations, we can see that, if the pH is below the pKa, then $K_0 \ll K_1$, K_2, K_3, which will push the equilibrium forward above the Henderson–Hasselbach result to increase the fraction of dimers, trimers and 4-mers present.

3.1 Interplay between pH and metal valency

Without forming the full free energy equation, we are able to use this modified Henderson–Hasselbach equation to examine the interplay between pH and metal valency. Indeed, if we assume, to test the impact of additional associations, that $K_1 = K_2 = K_3 = 1000$, and examine how the charge sparsity of the polyelectrolytes varies with pH under different conditions, we obtain Supplementary Fig. 28.

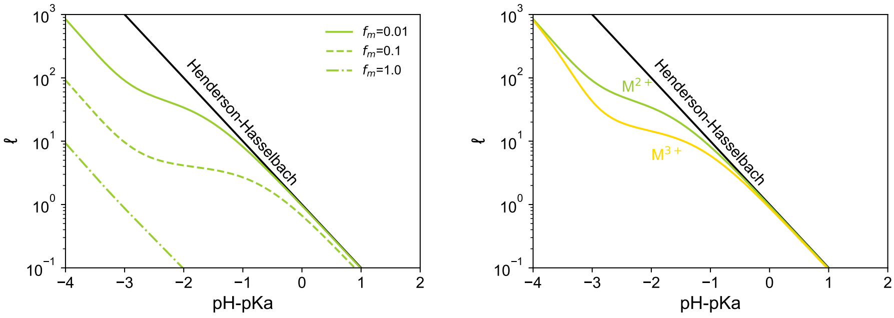

(a) (b)

Supplementary Fig. 28: Charge sparsity of a polyelectrolyte chain at **a,** different divalent metal concentrations and **b,** different valency metals at the same concentration, $f_m = 0.01$.

If we consider Supplementary Fig. 28a for a moment, we can see that the introduction of metal ions has lead to a downwards shift of the charge sparsity curve at low pH. This is expected as, given the equilibrium for associating with metal ions is so strong, whatever metal ions are present will force the deprotonation equilibrium forward such that there are enough charged sites to bind all charged sites. However, as we reach high pH where there are now more charged sites than metal ions, K_0 now becomes the determinant equilibrium constant where all curves eventually converge towards the Henderson–Hasselbach equation. This occurs until the metal ion concentration

becomes equal to the polymer bead concentration ($f_m = 1$); at this point, there will always be enough metal ions to bind all polyelectrolyte beads, leading to a constant offset from the Henderson–Hasselbach equation.

If we next consider the impact of the metal valency in Supplementary Fig. 28b, we can see that the ability to form a 4-mer has allowed the trivalent system to maintain a lower sparsity at the same pH as the divalent system. This would make sense as, with a fourth association, the deprotonation equilibrium is pushed further forward. The exceptions to this are at the very low and very high pH regime. As mentioned previously, at very low pH, the equilibrium is dominated by the metal ion associations. However, as there are so few charged sites, these metal ions are only able to associate once, meaning that the dominating equilibrium constant is K_1, which is identical in both systems. At very high pH, like the divalent system, we now have more charged sites than metal ions, leading to the deprotonation equilibrium dominating. All of these observations are commensurate with prior work using Monte Carlo simulations [18, 19].

4 Vibrational Spectroscopy for Study of Carboxyl/Carboxylate Association

The polyacid nature of MPECs gives spectral character of both Carboxyl (R-COOH) and Carboxylate (R-COO^-). The characteristic vibrational bands of the R-COOH/COO^-groups are in the range of 1000-2000cm^{-1} of the infrared at pH $\lesssim 2.5$ (acidic): the R-COOH carbonyl stretch shows a signature absorption at ∼1760cm^{-1} (monomer) / ∼1700cm^{-1} (dimer) with the alcohol C-OH stretch at ∼1240cm^{-1}, while carboxylate anion exhibits clear bond-and-a-half character with an asymmetric stretch at ∼1545cm^{-1} and a symmetric stretch at ∼1410cm^{-1}. Shown in Supplementary Fig. 29, pure PAA — without the presence of metal nitrate salts and sodium hydroxide buffer — only exhibit C=O stretch and C-O stretches, as expected, indicating the polymer is completely protonated. The positions of these characteristic bands are largely unaffected by the addition of NaOH for pH control, though the relative intensity of the acid (R-COOH) to conjugate base (R-COO^-) naturally changed owing to deprotonation of the carboxyl group.

4.1 Carboxylate Metal Coordination

While it is theoretically possible to ascertain metal ion in carboxylate bonding from its infrared spectrum, metal-oxygen vibrational modes fall below the mid-infrared spectrometer range as bond stiffness is inversely proportional to reduced mass ($k \propto \sqrt{\mu}$). The low symmetry of carboxylate ions further complicates determinations of carboxylate coordination by precluding the number of infrared or Raman active modes as a basis for structural determination. However, the type of carboxylate complexes and the coordination may be determined through secondary means owing to its effect on the the carbon-oxygen stretching frequencies.

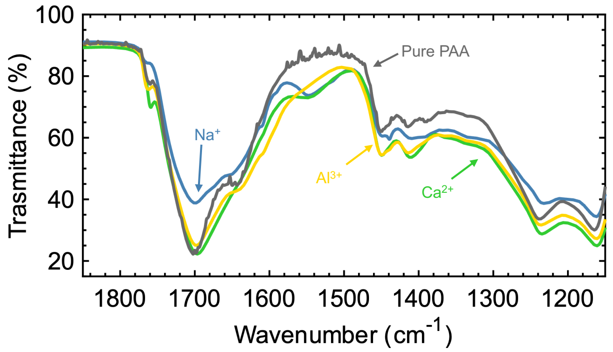

Supplementary Fig. 29: **Infrared spectra of PAA, Na^+, Ca^{2+}, and Al^{3+} gels.** Clear symmetric and asymmetric carboxylate modes are visible, while in in the case of pure PAA only the monomeric and dimerized carboxyl characteristic bands are present.

The work of Deacon and Phillips as well as Kirwan, Fawell, and Bronswijk have demonstrated the use of the relative intensities and separation ($\Delta\nu$) of the symmetric (ν_s) and asymmetric (ν_{as}) stretching frequencies of the carboxylate functional group to determine the bonding mechanisms: monodentate, chelating vs. bridging bidentate, and higher order coordination [20–22].

PAA is known to be neutralized between a pH range of 3-9 [22, 23], whereas pH $\gtrsim$ 9 (alkaline) groups are heavily ionized. The reference Sodium Polyacrylate salt for spectral analysis of complexation with higher valency metal cation species was taken at pH = 13, with $\Delta\nu_0 = 154\text{cm}^{-1}$. The presence of Carboxylate anion symmetric and asymmetric modes under acidic conditions — where pure PAA would be otherwise is almost entirely protonated — is indicative of their association with complexation of metal cations. The separation between these modes at pH $\lesssim$ 2.5 was substantially reduced relative to the reference ionic case, with $\Delta\nu = \{138.62, 141.30, 138.37\}\text{cm}^{-1}$ for Na^+, Ca^{2+}, Al^{3+}, respectively. Na^+, Ca^{2+}, and Al^{3+} exhibit $\Delta\nu < \Delta\nu_0$ is consistent with local coordination involving bidentate chelation or bridging structures between carboxylate ligands. The relatively high intensity of the asymmetric stretching mode for these complexes below the pKa of the carboxyl group suggests complexation. When taken together with $\Delta\nu < \Delta\nu_0$ and the intentional selection of cations of a hard ionic nature, this indicates a bidentate chelation complementary of the results of Kirwin et al. [21, 22].

Note that factors affecting these conclusions include the potential for a combination of bidentate coordination and monodentate pseudo-bridging where one oxygen of the carboxylate coordinates to the metal and the remaining oxygen is hydrogen-bonded to another ligand (carboxylate, water, or perhaps even residual nitrate) and anion exchange of the form

$$nRCO_2^- \cdot M^{n+} + nNa^+ \cdot (NO_3^-) \rightleftharpoons nRCO_2^- \cdot Na^+ + M^{n+} \cdot (NO_3^-)_n$$

may yield similar results to the case of sodium.

4.1.1 Quantum DFT Simulation of IR Spectra for Metal-Carboxylate Coordination

The complexation environment of metal cations was simulated using Quantum DFT methods, as described in **Section** 1. From these simulations, vibrational spectra and associated lowest energy coordinating geometries were produced allowing comparison with experimental findings. Energy minimization showed clear distinct coordination geometries between metal bonding based on valence, which was affected further as a result of competitive inclusion of water ligands in the crystal field of the cation, as captured in Supplementary Fig. 30. Representative structural models of coordination show clear bidentate chelation of the cations in vacuo, while introducing explicit water in the ligand field appears to introduces some monodentate chelation to otherwise predominantly bidentate chelation, seemingly consistent with experimental findings. These simulations, which are intentionally absent other species in MPEC gels (e.g., glycerol), are in generally good agreement with asymmetric and symmetric stretching modes of carboxylates seen experimentally: $\Delta\nu_0^{\mathrm{DFT}}$ values for Na^+, Ca^{2+}, and Al^{3+} corresponding to $116.04\mathrm{cm}^{-1}$, $77.36\mathrm{cm}^{-1}$ and $76.02\mathrm{cm}^{-1}$, respectively. This provided us with sufficient confidence to consider the binding energies obtained from these calculations to be somewhat representative of the real system.

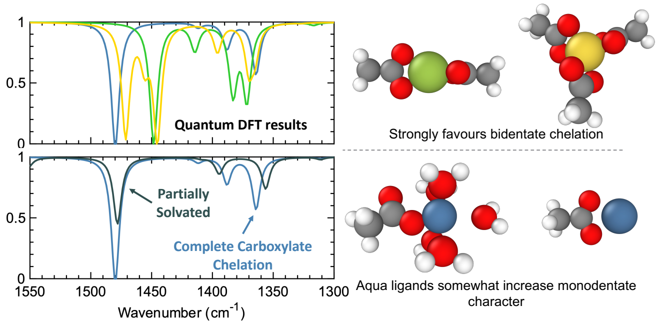

Supplementary Fig. 30: **Quantum DFT simulated infrared spectra of acetate ($RCOO^-$) complexation of Na^+, Ca^{2+}, and Al^{3+} in vacuo and with explicit coordinating aqua (H_2O) ligands.** Simulations demonstrate agreement with asymmetric and symmetric stretching modes of carboxylates seen experimentally. Representative structural models of coordination generated by simulation show a clear bidentate chelation coordinating environment in vacuo, while increasing explicit water in the ligand field appears to introduces some monodentate chelation to otherwise predominantly bidentate metal cation chelation, in agreement with experimental conclusions.

4.2 Effect of pH on Hydrogen Bonding in Poly(acrylic acid) Gels

Short-range hydrogen bonding networks are a well known mechanism of secondary bonding in polyacids, particularly poly(acrylic acid) (PAA) with repeating carboxylic acid pendant groups. The carboxyl groups in routinely dimerize, exhibiting local hydrogen bonding which increases inter-chain network strength, therefore the concentration of the carboxyl dimer relative to other absorbing moieties serves as a gauge of the extent of hydrogen bonding of the PAA polymer[5]. The extent of dimerization of the carboxyl and emergence of carboxylate groups was assessed in pure PAA samples as a function of increasing pH using a sodium hydroxide (NaOH) base.

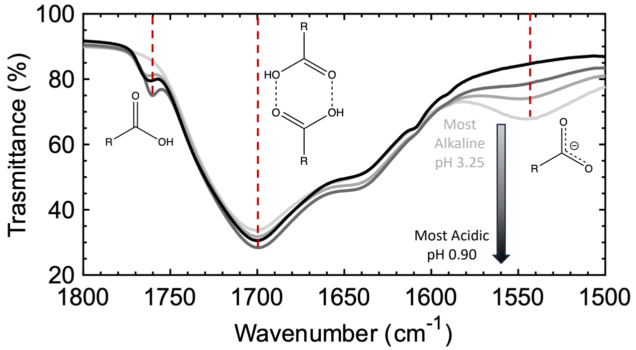

Supplementary Fig. 31: **Infrared spectrum of poly(acrylic acid) under variable pH from most acidic (pH ≈ 0.9) to most alkaline (pH ≈ 3.25) in region of signature bands for the carboxyl and asymmetric carboxylate stretches.** pH control of (de)protonation of the polyacid is clearly observed in the infrared spectrum via the disappearance of the monomeric R-COOH shoulder and emergence of the R-COO$^-$ asymmetric mode as pH increases.

This analysis allowed decoupling of the effects of pH changes on polymer charge sparsity from the potential decline in hydrogen bonding. Shown in Fig. 31, one thousand twenty four (1024) scans at 0.1cm^{-1} resolution were run from 1400^{-1} to 1900^{-1} for each sample at a given pH. Between six and ten samples (n = 6-10) we repeated for each pH step to generate meaningful statistics over the range. In each case, background corrected FTIR spectra of for these gels were deconvoluted and integrated using Fityk© to obtain a relative sense of the concentration of absorbing functional groups, ie. monomeric carboxyl (∼1760cm^{-1}), dimerized carboxyl (∼1700cm^{-1}), asymmetric stretching of carboxylate (∼1545cm^{-1}), and gylercol (∼1650cm^{-1}) [24]. Gaussian and

[5] Note: this analysis does not account for longer-range or multi-molecule hydrogen bonding mediated by water.

log normal functions (owing to the asymmetry of the vibrational well) were consistently used to model transmission troughs (absorption peaks) across the experimental pH range. Following consistent baseline correction, each spectrum was fitted within the subdomain of $\sim 1450^{-1}$ to $\sim 1850^{-1}$ using the popular non-linear least squares Levenberg-Marquardt algorithm. Fits were accepted provided weighted sum of squared residuals $\chi^2 \leq 5\%$, with weights based on standard deviations, $w_i = 1/\sigma_i^2$:

$$\chi^2(\mathbf{a}) = \sum_{i=1}^{N} \left[\frac{y_i - y(x_i;\ \mathbf{a})}{\sigma_i} \right]^2 = \sum_{i=1}^{N} w_i \left[y_i - y(x_i;\ \mathbf{a}) \right]^2$$

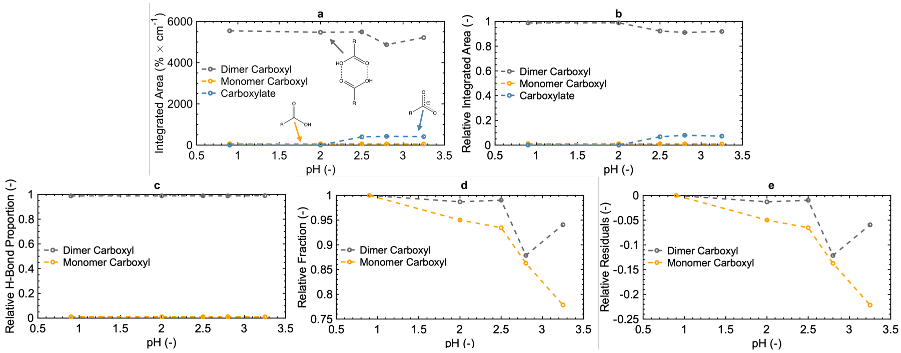

Supplementary Fig. 32: **Effect of pH change on extent of Hydrogen bonding in PAA gels, measured using FTIR.** Total integrated area of Transmission IR spectra (a) and relative integrated area (b) shows, within measurement uncertainty (n = 6-10), that carboxyl dimerization is unchanged, while monomer carboxyl groups decrease and carboxylate formation increases with increasing alkalinity for pH≤pKa of carboxyl groups. (c) Relative proportion of carboxyl groups participating in hydrogen bonding (dimerized), excluding ion-ion/ion-dipole interactions of carboxylate, show stability across experimentally explored pH range. (d) Fluctuation in dimerized and monomeric carboxyl pendant groups relative to most acidic (protonated) case show stability within uncertainty of measurement, with (e) residuals demonstrating small deviations in mean value with increasing pH for hydrogen bonded carboxyl groups and more significant deviation in non hydrogen bonded monomeric carboxyl groups suggesting initial disappearance of monomeric carboxyls may be tied to appearance of metal-bound carboxylate complexes.

The analytics summarized in Fig. 32 show that, within uncertainty, there is no net effect of pH change on the concentration (integrated area) of dimerized vs monomeric carboxyl functional groups within the limited range of $0.9 \lesssim \text{pH} \lesssim 3.5$. Similarly, after accounting for the emergence of carboxylate

functional groups owing to displacement of a proton by a metal cation (see **SI Section** 3), the extent of Hydrogen bonding in PAA gels is unchanged with 98.91% of carboxyl moieties dimerized and 1.09% monomeric across the tested pH range as shown in Supplementary Table 7. Monomeric carboxyl groups decrease and carboxylate formation increases with increasing alkalinity for pH≤pKa of carboxyl groups (pKa= 4.54). This is consistent with expectations from solution buffering theory, where proximity to the pKa determines the difficulty of deprotonation and thereby the extent of it. Considering all samples in this study were pH< 4.54, as outlined in Supplementary Table 3, a highly protonated polyacid is the expectation, with the deprotonated sites being nearly exclusively those complexed with metal cations.

Integrated areas for each dimerized and monomeric carboxyl absorption were normalized by the most acidic (protonated) case to examine relative trends. This analysis showed that within measurement uncertainty there is again stability in the dimer and a decreasing trend in the monomer. Residuals for this analysis demonstrated small deviations in mean value with increasing pH for hydrogen bonded carboxyl groups and more significant deviation in the non hydrogen bonded monomeric carboxyl groups suggesting that, perhaps, the initial disappearance of monomeric carboxyls may be tied to the early appearance of metal-bound carboxylate complexes before the break-up of R-COOH dimers for metal cation complexation.

No. Samples	pH	Dimer Area	Dimer StDev	Monomer Area	Monomer StDev	Carboxylate Area	RCOO- StDev	Total Area	[HTML]333333	Total Uncertainty
10	0.9	5545.51	±444.52	64.82	±15.01			5610.33	[HTML]333333	±444.77
6	2	5473.56	±362.07	61.59	±7.64			5535.15	[HTML]333333	±362.15
10	2.54	5491.60	±148.89	60.59	±4.00	398.01	±151.55	5950.20	[HTML]333333	±212.49
9	2.8	4872.06	±644.55	55.92	±8.55	423.39	±200.23	5351.37	[HTML]333333	±674.99
10	3.25	5216.37	±447.09	50.45	±23.78	410.33	±140.15	5677.15	[HTML]333333	±469.14

Supplementary Table 5: **Change in integrated areas of carboxyl and carboxylate infrared modes in PAA gels owing to pH.** Integrated areas of each absorption band as a function of increasing pH of the recursor resin. All areas and their standard deviations (or propagated uncertainties) are reported in units of $cm^{-1} \cdot \%$ corresponding to integration of percent transmission with respect to wavenumber.

pH	Dimer Content	Monomer Content	Carboxylate Content
0.9	98.84%±11.14%	1.16%±0.28%	0.00%±0.00%
2	98.89%±9.20%	1.11%±0.16%	0.00%±0.00%
2.54	92.29%±4.14%	1.02%±0.11%	6.69%±2.60%
2.8	91.04%±16.64%	1.05%±0.18%	7.91%±3.79%
325	91.88%±10.94%	0.89%±0.42%	7.23%±2.53%

Supplementary Table 6: **Proportion of infrared absorption as a percent of total absorption from all carboxyl and carboxylate groups.**

$$\phi_{\mathrm{i}} = \begin{cases} \frac{\int_{RCOOH_d}}{\int_{RCOOH_d} + \int_{RCOOH_m} + \int_{RCOO^-}} \\ \frac{\int_{RCOOH_m}}{\int_{RCOOH_d} + \int_{RCOOH_m} + \int_{RCOO^-}} \\ \frac{\int_{RCOO^-}}{\int_{RCOOH_d} + \int_{RCOOH_m} + \int_{RCOO^-}} \end{cases}$$

Uncertainties reflect propagated error from standard deviations among samples as shown in Supplementary Table 5 and error gained among sum of total absorption from all bands of interest.

pH	Degree of Dimerization	Uncertainty in Degree of Dimerization	Degree Monomeric	Uncertainty in Monomeric
0.9	98.84%	11.33%	1.16%	0.28%
2	98.89%	9.30%	1.11%	0.16%
2.54	98.91%	3.82%	1.09%	0.08%
2.8	98.87%	18.51%	1.13%	0.23%
3.25	99.042%	12.27%	0.96%	0.46%

Supplementary Table 7: **Degree of carboxyl groups readily available for Hydrogen bonding.** Carboxyl groups are capable of forming strong hydrogen bonding networks in PAA owing to their capacity to dimerize. The relative proportion of all carboxyl species which are dimerized vs. in monomeric forms is presented as a total of all carboxyls, $\phi_{\mathrm{d}} = \frac{\int_d}{\int_d + \int_m}$ and $\phi_{\mathrm{m}} = \frac{\int_m}{\int_d + \int_m}$ Uncertainties reflect propagated error from standard deviations among samples as shown in Supplementary Table 5 and error among sum of absorption from all carboxyl bands.

pH	Degree of Dimerization	Dimerization Residuals	Degree Monomeric	Monomeric Residuals
0.9	100%±0.00%	0.00%±0.00%	100%±0.28%	0.00%±0.00%
2	98.70%±10.26%	-1.30%±10.26%	95.02%±24.96%	-4.98%±24.96%
2.54	98.03%±8.38%	-0.97%±8.38%	93.47%±22.51%	-6.53%±22.51%
2.8	87.86%±13.59%	-12.14%±13.59%	86.28%±23.94%	-13.72%±23.94%
325	94.06%±11.04%	-5.94%±11.04%	77.83%±40.88%	-22.17%±40.88%

Supplementary Table 8: **Proportion of infrared absorption from all carboxyl groups relative to most acidic reference case and residuals.** To gauge relative change of degree of dimerization of monomeric carboxyl groups, the total integrated absorption from these bands at each pH is expressed as a percent of the maximal case (most acidic), $\phi_{\mathrm{i}} = \frac{i}{i_0}$ Shown adjacent is the residuals in each case and their associated uncertainties, $\mathrm{e}\left(\phi_{texti}\right) = \frac{(i-i_0)}{i_0}$ Uncertainties in each case reflect propagated error from measurement standard deviations as shown in Supplementary Table 5 among samples and error gained from the above operations.

5 Mean-field theory of multivalent ions and polyelectrolyte systems

The theory we develop to model multivalent ions and polyelectrolyte systems accounts for three effects: 1. excluded-volume effects, 2. long-range electrostatic effects and 3. short-range association between charged sites on the polyelectrolyte backbone and the metal crosslinkers. The resulting representation resembles that shown in Supplementary Fig. 33. The total free energy (no longer free energy density) for this system is then expressed as:

$$F = F_{\mathrm{id.}} + F_{\mathrm{exc.}} + F_{\mathrm{ele.}} + F_{\mathrm{assoc.}}\,. \tag{S.20}$$

5.1 Excluded Volume Effects

To account for the excluded volume contribution, $F_{\mathrm{exc.}}$, we take inspiration from the Statistical Associating Fluid Theory (SAFT), developed by Chapman et al. [25], where one treats species as fused chains of m hard-sphere monomers with beads of diameter σ. This hard-chain contribution to the free energy is obtained from:

$$\frac{F_{\mathrm{exc.}}}{Nk_{\mathrm{B}}T} = \bar{m}\frac{F_{\mathrm{HS}}}{Nk_{\mathrm{B}}T} - \sum_i x_i(m_i-1)\ln[g_{\mathrm{HS}}(\sigma_i)]\,, \tag{S.21}$$

where x_i, m_i and σ_i are the mole fraction, number of beads and bead diameter of species i, respectively. $\bar{m}$ is the molar-average number of segments in the

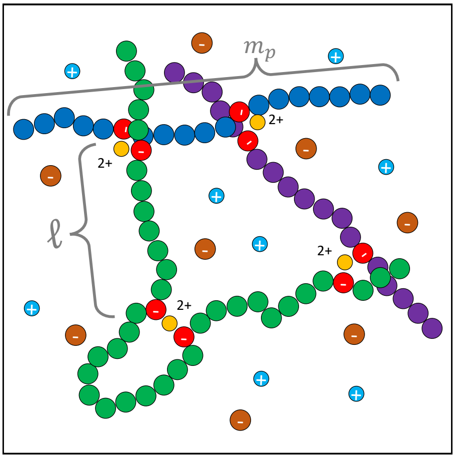

Supplementary Fig. 33: Visual representation of the proposed theory. Colors have been used to distinguish between chains. Charged sites on the polymer backbone have been highlighted in red. The metal crosslinker, polyelectrolyte counter-ion and metal co-ion have been highlighted in yellow, light blue and brown, respectively.

system:

$$\bar{m} = \sum_i x_i m_i \,. \tag{S.22}$$

Note that the symbol N will now refer to the total number of particles in the system, rather than polymer chain length. The hard-sphere contribution to the free energy, F_{HS}, is obtained from the Carnahan-Starling equation of state [26]:

$$\frac{F_{\mathrm{HS}}}{Nk_{\mathrm{B}}T} = \frac{1}{\zeta_0}\left[\frac{3\zeta_1\zeta_2}{(1-\zeta_3)} + \frac{\zeta_2^3}{\zeta_3(1-\zeta_3)^2} + \left(\frac{\zeta_2^3}{\zeta_3^2} - \zeta_0\right)\ln(1-\zeta_3)\right] , \tag{S.23}$$

where the packing fractions, ζ_n, are obtained from:

$$\zeta_n = \frac{\pi}{6}\rho\sum_i x_i m_i \sigma_i^n , \quad n \in 0,1,2,3 \,, \tag{S.24}$$

where ρ is the total number density (N/V) of the system. The hard-sphere pair distribution function, $g_{\mathrm{HS}}(\sigma_i)$, is also obtained using the Carnahan-Starling equation of state:

$$g_{\mathrm{HS}}(\sigma_i) = \frac{1}{1-\zeta_3} + \frac{\sigma_i}{2}\frac{3\zeta_2}{(1-\zeta_3)^2} + \frac{\sigma_i^2}{4}\frac{2\zeta_2^2}{(1-\zeta_3)^3} \,. \tag{S.25}$$

We note that, unlike Zhang et al. [27], we only use the hard-sphere pair distribution function in equation S.21 to model the formation of chains, despite the presences of charges in the chain. In Zhang et al.'s work [27], all beads in

the chain are charged whereas, in our work, the charged beads are separated by ℓ uncharged beads. As such, the beads in direct contact with one another will only interact via hard-sphere interactions. However, if the spacing between charged beads is reduced to the point of being below the screening length of the system, long-range interactions within the chain will play a role. For this reason, we will avoid such low charge sparsities.

5.2 Electrostatic Effects

The electrostatic contribution, $F_{\text{ele.}}$, is obtained using the Debye–Hückel equation [28], given by:

$$\frac{F_{\text{ele.}}}{Nk_{\text{B}}T} = -\frac{\rho}{3} l_{\text{B}} \kappa \sum_i x_i f_i Z_i^2 \chi(\kappa\sigma_i)\,, \tag{S.26}$$

where f_i and Z_i are the number and valency of charges on species i. l_{B} is the Bjerrum length:

$$l_{\text{B}} = \frac{\beta e_c^2}{4\pi\epsilon_0\epsilon_{\text{r}}}\,, \tag{S.27}$$

where e_c is the charge of an electron, ϵ_0 is the permittivity of free space and ϵ_{r} is the dielectric constant of the medium. The Debye inverse screening length, κ, is given by:

$$\kappa^2 = 4\pi l_{\text{B}} \rho \sum_i x_i f_i Z_i^2\,. \tag{S.28}$$

The function $\chi(y)$ can be obtained from:

$$\chi(y) = \frac{3}{y^3}\left[\frac{3}{2} + \ln(1+y) - 2(1+y) + \frac{1}{2}(1+y)^2\right]. \tag{S.29}$$

We note that a few approximations are made when using the Debye–Hückel equation. Firstly, as part of the derivation for the Debye–Hückel equation, we assume that we are in a regime where the inverse screening length is large. Silva et al. [29] have shown that, even when this approximation is not made and the Poisson–Boltzmann expression is solved in full, the differences between this and the Debye–Hückel equation remain minor up to high salt concentrations. One limitation of this is when considering that charges on the polymer backbone separated by ℓ monomers. In the case of a freely jointed chain, so long as the distance between monomers exceeds the screening length ($\kappa\ell\sigma \gg 1$), it is still valid to use the Debye–Hückel expression in this context. Following a similar line of thought, we also assume that our solvent, that is, the dielectric medium, can be treated implicitly.

Finally, as done in the work by Zhang et al. [27] and, more recently, Ylitalo et al. [30], one could also capture electrostatic effects using Blum [31] implicit mean-spherical approximation (MSA). Based on the work by Maribo-Mogensen et al. [32], we note that the differences between the MSA and Debye–Hückel expression can be accounted for using a simple rescaling of the

bead diameter, σ_i. As such, given we are aiming to analyse this system qualitatively and that the Debye–Hückel term is less computationally demanding, we shall use this term instead.

5.3 Association Effects

When modelling the association between the metal crosslinker and charged site on the polyelectrolyte backbone, we assume that the number of sites on the metal crosslinker is equal to its valency ($Z_m = f_m$). That is, it cannot crosslink with more charged sites than its charge can compensate for; this would be intuitive as, once the crosslink has become locally neutral, it will be energetically unfavorable to crosslink with an additional charge on the backbone. As for the polyelectrolyte, we will treat each charged site as an association site; given all charged sites are monovalent, each polyelectrolyte then has f_p association sites.

At this point, we note that the theory developed by Semenov and Rubinstein [33] can be reproduced using the first-order Wertheim Thermodynamic Perturbation Theory (WTPT1) in the case where we assume the binding energy is large and the fluid is ideal ($g(r) \approx 1$). Given we are now modelling the chain as having both excluded volume and electrostatic effects, this approximation is no longer appropriate. As such, we will now rigorously re-derive the association contribution, $F_{\text{assoc.}}$. We start with a system where no sites are associated; in this case, we define the local number density of the sites as:

$$\rho_i(\mathbf{r}_i) = \frac{N_i(\mathbf{r}_i) f_i}{V}\,, \tag{S.30}$$

where $N_i(\mathbf{r}_i)$ is the local number density of species i at the position vector $\mathbf{r}_i$. We now assume that some of these sites have been associated such that the local number density of sites not associated, $\rho_{i0}(\mathbf{r}_i)$, is given by:

$$\rho_{i0}(\mathbf{r}_i) = \frac{N_i(\mathbf{r}_i) f_i}{V} X_i(\mathbf{r}_i)\,, \tag{S.31}$$

where $X_i(\mathbf{r}_i)$ is the local fraction of sites on species i not associated. As a result, the free energy change of these sites associating is given by:

$$\begin{aligned}\frac{F_{\text{assoc.}}}{k_{\text{B}}T} = &\sum_i \int \rho_i(\mathbf{r}_i) \ln\left(\frac{\rho_{i0}(\mathbf{r}_i)}{\rho_i(\mathbf{r}_i)}\right) + \rho_i(\mathbf{r}_i) - \rho_{i0}(\mathbf{r}_i) d\mathbf{r}_i \\ &- \int\int \rho_{p0}(\mathbf{r}_p)\rho_{m0}(\mathbf{r}_m) g(r_{pm}) f(r_{pm}) d\mathbf{r}_p d\mathbf{r}_m\,,\end{aligned} \tag{S.32}$$

where the indices p and m refer to the polyelectrolyte and metal sites, and r_{pm} is the distance between the polyelectrolyte and metal sites. $g(r)$ is the pair distribution function and $f(r)$ is the Mayer-f function given by:

$$f(r) = [\exp -\beta\phi(r) - 1]\,, \tag{S.33}$$

where $\phi(r)$ is the potential characterising the association interaction. To simplify our expression for the free energy, we will assume the system is homogeneous (that is our number densities have no positional dependence), giving:

$$\frac{F_{\text{assoc.}}}{k_{\text{B}}T} = \sum_i \left[\rho_i \ln\left(\frac{\rho_{i0}}{\rho_i}\right) + \rho_i - \rho_{i0}\right] \int d\mathbf{r}_i \\ - \rho_{p0}\rho_{m0} \int\int g(r_{pm}) f(r_{pm}) d\mathbf{r}_p d\mathbf{r}_m \,, \tag{S.34}$$

$$\frac{F_{\text{assoc.}}}{k_{\text{B}}T} = \sum_i N_i f_i \left[\ln X_i + 1 - X_i\right] \\ - 4\pi\rho_{p0}\rho_{m0} V \int g(r_{pm}) f(r_{pm}) r_{pm}^2 dr_{pm} \,, \tag{S.35}$$

$$\frac{F_{\text{assoc.}}}{Nk_{\text{B}}T} = \sum_i x_i f_i \left[\ln X_i + 1 - X_i\right] - \rho x_p x_m f_p f_m X_p X_m \Delta_{pm} \,. \tag{S.36}$$

For clarity, we have defined the association strength between the polyelectrolyte and metal, Δ_{pm}, as:

$$\Delta_{pm} = 4\pi \int g(r_{pm}) f(r_{pm}) r_{pm}^2 dr_{pm} \,. \tag{S.37}$$

If we now minimise equation S.36 with respect to all X_i, we obtain:

$$\frac{\partial}{\partial X_p}\left(\frac{F_{\text{assoc.}}}{Nk_{\text{B}}T}\right) = x_p f_p \left[\frac{1}{X_p} - 1\right] - \rho x_p x_m f_p f_m X_m \Delta_{pm} = 0 \,, \tag{S.38}$$

$$\frac{\partial}{\partial X_m}\left(\frac{F_{\text{assoc.}}}{Nk_{\text{B}}T}\right) = x_m f_m \left[\frac{1}{X_m} - 1\right] - \rho x_p x_m f_p f_m X_p \Delta_{pm} = 0 \,, \tag{S.39}$$

which we can re-arrange to give a set of mass-action equations:

$$X_p = \frac{1}{1 + \rho x_m f_m \Delta_{pm} X_m} \,, \tag{S.40}$$

$$X_m = \frac{1}{1 + \rho x_p f_p \Delta_{pm} X_p} \,. \tag{S.41}$$

Substituting these expressions to equation S.36 will give:

$$\frac{F_{\text{assoc.}}}{Nk_{\text{B}}T} = \sum_i x_i f_i \left[\ln X_i + \frac{1 - X_i}{2}\right] \,. \tag{S.42}$$

At this stage, we note the similarities between equation S.42 and the sticker

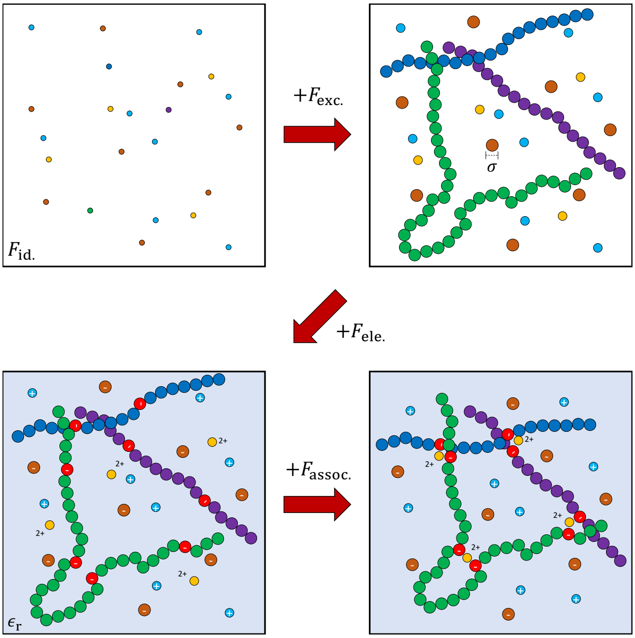

Supplementary Fig. 34: Summary of the impact of each term in the proposed theory. The dielectric medium is represented with a change in the background color. Color scheme is identical to that in Supplementary Fig. 33.

contribution from Semenov and Rubinstein [33]'s work. Indeed, if we had allowed the polyelectrolyte sites to associate directly with each other and defined $p = 1 - X_p$, the above expression would reduce to that of Semenov and Rubinstein [33], with one key difference. Our association strength, given in equation S.37 now requires information from the pair distribution function of our reference system (charged hard-spheres). We will assume that the association between sites is characterised by Coulomb's potential such that:

$$\phi(r_{pm}) = \frac{l_{\mathrm{B}} Z_m}{r_{pm}}\,. \tag{S.43}$$

Expressions for the pair distribution function between charged species do exist, as obtained from Blum's implicit MSA model [34], which was further simplified by Jiang et al. [35] to what is known as the exponential approximation of the implicit MSA pair distribution function ($g^{\mathrm{EXP}}(r)$). However, the integral in equation S.37 is not trivial and would be numerically expensive to evaluate. Typically, within SAFT-type approaches, this is dealt with by either developing correlations for the association strength [36], or by treating the pair distribution function and Mayer-f function as constants (evaluated at contact). We can then introduce a fudge-factor (also known as the 'bonding

volume', $\kappa_{pm}\sigma_{pm}^3$) to approximate the association strength [25, 37]:

$$\Delta_{pm} = \kappa_{pm}\sigma_{pm}^3 g^{\mathrm{EXP}}(\sigma_{pm})\left[\exp\left(\frac{l_{\mathrm{B}}Z_m}{\sigma_{pm}}\right) - 1\right]. \tag{S.44}$$

This now gives us a full set of equations to describe our system. The development of this theory is summarised in Supplementary Fig. 34.

5.4 Definition of phase equilibrium

In the case where a phase split occurs between phases α and β at a specified density for each species, ρ_{i0}, and temperature, the total free energy density ($\Psi = F/V$) will be given by:

$$\Psi = \varphi\Psi_\alpha(\boldsymbol{\rho}_\alpha, T) + (1-\varphi)\Psi_\beta(\boldsymbol{\rho}_\beta, T), \tag{S.45}$$

where φ is the volume fraction of phase α and $\boldsymbol{\rho}_j$ is the vector containing the density of each species in phase j. In order to conserve the total density of each species, we must introduce the Lagrangian multiplier, μ_i, for each species. Furthermore, as we are now including charged species, we must ensure electroneutrality in all phases. Given the input densities must satisfy electroneutrality, then we only require one more Lagrangian multiplier, ψ, to ensure this will be satisfied. As a result, the total Lagrangian for this system is:

$$\begin{aligned} \ell = &\varphi\Psi_\alpha(\boldsymbol{\rho}_\alpha, T) + (1-\varphi)\Psi_\beta(\boldsymbol{\rho}_\beta, T) \\ &+ \sum_i \mu_i(\varphi\rho_{i\alpha} + (1-\varphi)\rho_{i\beta} - \rho_{i0}) \\ &+ \psi\varphi\sum_i \rho_{i\alpha} f_i Z_i \,. \end{aligned} \tag{S.46}$$

To determine the composition of each phase, we must minimise equation S.46 with respect to all densities (ρ_{ij}), volume fraction (φ), and all Lagrangian multipliers. In doing so, we find the following system of equations:

$$\mu_i = \frac{\partial\Psi_\alpha}{\partial\rho_{i\alpha}} + \psi f_i Z_i = \frac{\partial\Psi_\beta}{\partial\rho_{i\beta}}, \quad \forall i \tag{S.47}$$

$$0 = \Psi_\alpha - \Psi_\beta + \sum_i \mu_i(\rho_{i\alpha} - \rho_{i\beta}) + \psi\sum_i \rho_{i\alpha} f_i Z_i, \tag{S.48}$$

$$0 = \varphi\rho_{i\alpha} + (1-\varphi)\rho_{i\beta} - \rho_{i0}, \quad \forall i \tag{S.49}$$

$$0 = \sum_i \rho_{i\alpha} f_i Z_i \,. \tag{S.50}$$

As we can see above, the choice of notation for the Lagrangian multipliers was intentional as μ_i corresponds to the chemical potential of each species and ψ

corresponds to the electrochemical potential difference between phase α and β.

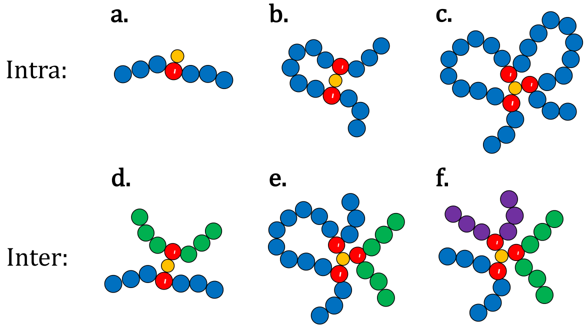

Supplementary Fig. 35: Different possible configurations for intra- (a-c) and inter- (d-f) crosslinks. Different colors represent different chains.

5.5 Determining fraction of interchain crosslinked sites

One aspect of Semenov and Rubinstein [33]'s work that hasn't been explored in much depth is, if one assumes that all sites are fully associated (i.e. $X_i = 1$) and that intra-crosslinks are only formed between nearest neighbours, then one can calculate the fraction of sites inter- and intra-crosslinked. In making these assumptions, we can assume that the entropic penalty for forming an intra-crosslink is [33]:

$$\Delta S_{\text{intra.}} = m_{\text{intra.}} k_{\text{B}} \ln \ell^{3/2} \,, \tag{S.51}$$

where $m_{\text{intra.}}$ is the number of intra-crosslinked pairs. If we assume our reference system is the fully associated system ($Z_{\text{assoc.}}$), then the partition function for the intra-crosslinked system is:

$$Z_{\text{intra.}} = Z_{\text{assoc.}} P_{\text{comb.}} W \exp\left(\Delta S_{\text{intra.}}/k_{\text{B}}\right) , \tag{S.52}$$

where $P_{\text{comb.}}$ and W are defined the same way as the dimer combinatorial term, only with $m_{\text{intra.}}$ being selected from m_t trimers. If we minimise equation S.52 with respect to $m_{\text{intra.}}$ and define $f_{\text{intra.}} = m_{\text{intra.}}/m_p$, then we obtain the following expression:

$$f_{\text{intra.}} = \frac{-1 + \sqrt{1 + 4\ell^{1/2}\rho_p \sigma_p^3}}{2\ell^{1/2}\rho_p\sigma_p^3} \,. \tag{S.53}$$

Springer Nature 2021 LaTeX template

The above equation is technically only valid for the divalent system as, for the trivalent system, there are a few more ways one could form interchain crosslinks. This is shown in Supplementary Fig. 35. As we can see, in the case of the trivalent system, the fraction of sites which have formed an intra-crosslink is given by:

$$f_{\text{intra.,tri}} = \left(f_{\text{intra.}} X_m^2 + f_{\text{intra.}}^2 X_m^3\right) \,. \tag{S.54}$$

Furthermore, in terms of forming interchain crosslinks, the trivalent system has three ways of doing so, rather than just one in the case of the divalent system:

$$\begin{aligned} f_{\text{inter.,tri}} &= (1 - f_{\text{intra.}})(1 - X_m)^2 X_m \\ &\quad + (2 f_{\text{intra.}}(1 - f_{\text{intra}}) + (1 - f_{\text{intra}})^2)(1 - X_m)^3 \,. \end{aligned} \tag{S.55}$$

6 Thermogravimetric Analysis of MPEC Gels

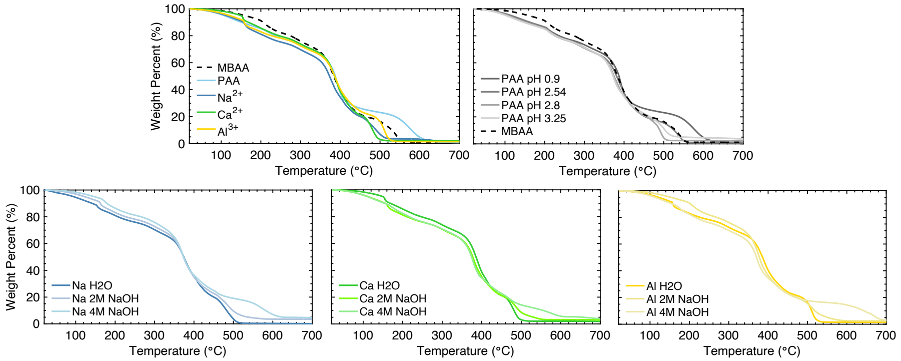

Supplementary Fig. 36: **Thermal Gravimetric Analysis (TGA) for MPEC gels for valency and pH.** Weight loss profiles of MPEC gels as a function of valence and pH, with comparison to pure PAA and MBAA reference. All samples heated from 20–1000°C at 5°C per minute under a steady 25mL per minute air. Within a common ion comparison, lighter colors correspond to increasing pH, as labelled.

Thermogravimetric Analysis (TGA) was used to precisely quantify the water content in our equilibrated gels. Measurements were made using inflection points in the TGA data in Figure 36 using the first derivative; two regions were identified from this based on change in slope corresponding to loosely bound water held in the structure and tightly bound (crystalline) water solvating the polymer and metal cation-carboxylate bonds. Start and end points for these measurements as well as the relative values are recorded in Table 9. Across all variations in metal ion types and system pH, our analysis consistently revealed that gels contain approximately $\sim 14\% \pm 2.14\%$ water by weight with a median of 14.27% water after post-processing. This reasonably

uniform water content ensures a fair and accurate comparison between gels, enabling us to effectively isolate the effects of valencies and pH.

Supplementary Table 9: **Tabulation of quantified gel water content from thermogravimetric (TGA) analysis.** Using inflection points in derivative data, regions of loosely bound water evaporation and tightly bound (crystalline) water that solvates the polymer/metal-carboxylate bonds was determined. The cumulative total of these values is reported as the total water content (wt%).

		**Loosely Bound**			**Crystalline**			**Total**
	pH	Start	End	Delta	Start	End	Delta	-
PAA	0.9	100%	84.04%	15.96%	84.04%	83.66%	0.38%	16.34%
PAA	2.54	100%	85.97%	14.03%	85.97%	85.64%	0.33%	14.36%
PAA	2.8	100%	85.04%	14.96%	85.04%	84.57%	0.47%	15.43%
PAA	3.25	100%	84.48%	15.52%	84.48%	83.42%	1.06%	16.58%
Na H_2O	2.55	100%	88.88%	11.12%	88.88%	85.96%	2.92%	14.04%
Na 2M NaOH	2.95	100%	91.48%	8.52%	91.48%	87.68%	3.81%	12.32%
Na 4M NaOH	3.25	100%	93.44%	6.56%	93.44%	88.83%	4.61%	11.17%
Ca H_2O	2.15	100%	94.90%	5.10%	94.90%	90.87%	4.03%	9.13%
Ca 2M NaOH	2.72	100%	89.77%	10.23%	89.77%	85.89%	3.88%	14.11%
Ca 4M NaOH	3.1	100%	89.07%	10.93%	89.07%	85.73%	3.34%	14.27%
Al H_2O	1.4	100%	91.64%	8.36%	91.64%	86.90%	4.75%	13.11%
Al 2M NaOH	2.4	100%	89.00%	11.00%	89.00%	84.35%	4.66%	15.65%
Al 4M NaOH	2.97	100%	89.17%	10.84%	89.17%	84.45%	4.71%	15.55%

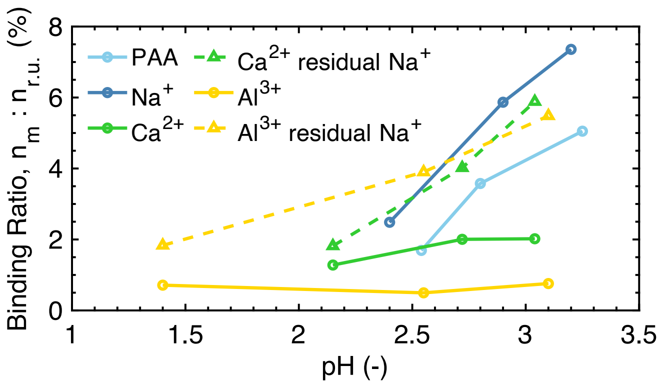

Supplementary Fig. 37: Metal cation (M^{n+}) to polyelectrolyte repeat unit (r.u./$RCOO^-$) binding ratio as inferred from TGA using residual oxide weights. Mass percent by difference was used to calculate residual metal oxide from soot and distinguish metal oxides of the target species (Na^+, Ca^{2+}, Al^{3+}) from excess counter-ion (Na^+) introduced as a result of pH regulation with NaOH base. Fraction of PAA were determined by conversion of the metal oxide content into equivalents of metal species, which when subtracted from the fully dehydrated weight (wt% at 200–260°C, depending on metal species) yielded weight fraction owed to PAA. Experimentally determined binding ratios for target species (≈1–2.5% at most acidic pH) are similar to theoretically expected ratios ($\lesssim$2% at most acidic pH) predicted based on resin composition and scale within expectations for pH changes, remaining well within correct order of magnitudes.

TGA was also used to compute the metal cation (M^{n+}) to polyelectrolyte repeat unit (r.u./$RCOO^-$) binding ratio using residual oxide weights as shown in Figure 37. Mass percent by difference was used to calculate residual metal oxide from soot and distinguish metal oxides of the target species (Na^+, Ca^{2+}, Al^{3+}) from excess counter-ion (Na^+) introduced as a result of pH regulation with NaOH base:

$$\mathrm{wt\%_{MOx}} = \mathrm{wt\%_{MPEC}} - \mathrm{wt\%_{Soot}}$$

$$n_{\mathrm{M}} = (\mathrm{wt\%_{MOx}}) \times \frac{1\mathrm{g}}{\mu_{\mathrm{MOx}}} \times \chi_{\mathrm{M,MoX}}$$

$$n_{\mathrm{r.u.}} = (\mathrm{wt\%_{PAA}}) \times \frac{1\mathrm{g}}{\mu_{\mathrm{AA}}}$$

$$r = \mathrm{M} : \mathrm{PAA} \cong \frac{n_{\mathrm{M}}}{n_{\mathrm{r.u.}}}$$

Where $\chi_{\mathrm{M,MoX}}$ represents the stoichiometric ratio of metal to metal oxide. In all cases, the most stable oxides at room temperature and pressure were assumed. Soot content ($\mathrm{wt\%_{Soot}}$) was determined by running pure PAA samples to 1000°C. Sodium incorporation from precursor SA was assumed consistent in all samples, enabling determination of excess sodium uptake, as

shown in the **Fig.** 36. Molar mass of the repeat unit was presumed to be approximately that of acetic acid, rather than acetate, owing to the presumption of disproportionately protonated carboxyl groups because pH<pKa of the carboxylates. Fractions of PAA were determined by conversion of the metal oxide content into equivalents of metal species, which when subtracted from the fully dehydrated weight (wt% at 200–260°C, depending on metal species) yielded weight fraction owed to PAA:

$$\mathrm{wt\%_M} = n_M \times \mu_\mathrm{M} \times \frac{1}{\mathrm{1g}}$$

$$[\mathrm{wt\%_{Gel}} - \mathrm{wt\%_M}] \times \frac{\mathrm{1g}}{\mu_\mathrm{AA}} \approx n_{r.u.}$$

Experimentally determined binding ratios for target species (≈1–2.5% at most acidic pH) are similar to theoretically expected ratios ($\lesssim$2% at most acidic pH) predicted based on resin composition and scale within expectations for pH changes, remaining well within correct order of magnitudes.

7 Solvent effect on MPEC gels

Solvent is a crucial parameter significantly affecting material behavior. While we kept the solvent content constant across all samples in the main text to isolate other variables, we recognized the importance of understanding solvent effects on material properties. Therefore, we conducted additional thorough studies on the effect of solvents using both experimental and computational approaches.

7.1 Experimental results

7.1.1 Effect of Co-solvent on Mechanical Response

The two primary solvents of the fabricated MPEC gels are water and glycerol. According to TGA analysis in section 6 in the SI, all equilibrated gel presented in the main text, irrespective of the metal ions and the system pH chosen, contain ∼14% ± 2.14% water by weight. and 10 v/v% glycerol. To change the amount of water in the gels, we varied the amount of water by placing the printed-equilibrated MPEC gels in the high-humidity (RH∼80%) environment. Figure 38a confirms that gels contain different amount of water by hydrating them different amount. The prepared gels were characterized by quasi-static tensile testing. Loading and unloading were applied to the strain of 2.5 and the measured modulus and hysteresis were supplied in the Figure 38b. Increasing water content from 10% to 20% and 30% weight results in a reduction of modulus by a factor of 1.41 and 4.80 for Al-MPEC and 1.86 and 3.92 for Ca-MPEC, as observed in tensile tests.

Glycerol contents can also be varied by introducing different amount into the photo resin.. In decreasing the glycerol content, although this is compensated by additional water, as water is more volatile than glycerol, the total

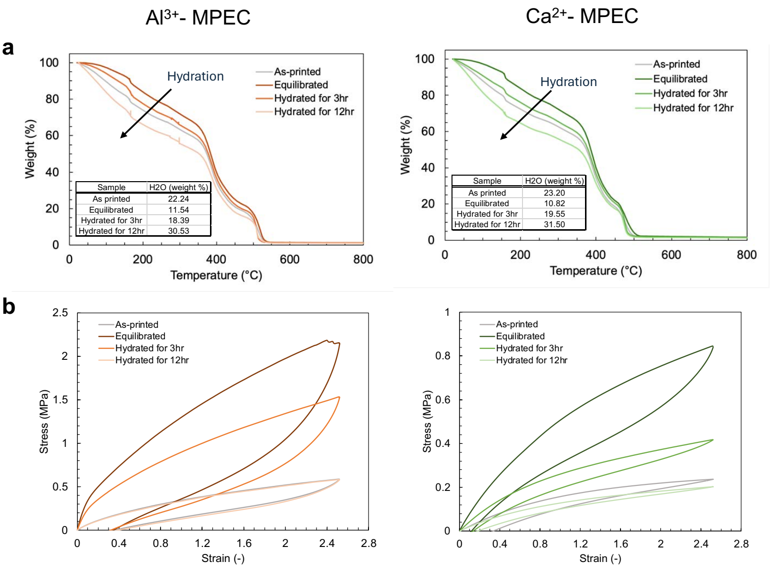

Sample	Modulus (MPa)	Area under the curve, W (MJ/m3)	W/Wo (%)
As-printed	0.576	0.407	43.994
Equilibrated	2.706	2.014	57.275
Hydrated, 3hr	1.921	1.308	53.687
Hydrated, 18hr	0.564	0.425	46.877

Sample	Modulus (MPa)	Area under the curve, W (MJ/m3)	W/Wo (%)
As-printed	0.232	0.103	25.593
Equilibrated	0.830	0.408	30.362
Hydrated, 3hr	0.447	0.131	19.268
Hydrated, 18hr	0.212	0.054	16.205

Supplementary Fig. 38: Different amount of water was included in trivalent (left) and divalent (right) MPEC gels by equilibrating the gels in high-humidity environment (RH∼80%). **a** TGA analysis confirms that the gels contain different amount of water contents. **b** Stress-strain curves obtained for different water contents on. The respective young's moduli, area enclosed by the loading-unloading curve (hysteresis) and energy dissipation percent (hysteresis normalized by the total energy induced from the applied loading) of each gel have been given in the tables above.

remaining solvent after drying is decreased, reducing the overall free volume of the gel. The impact of this reduction is demonstrated in figure. 39.

As we can see, in the case of both the divalent and trivalent gels, reducing the glycerol fraction results in stiffer gels. Furthermore, the gels typically fail much earlier with a smaller glycerol fraction. The exception to this is the 5 v/v% glycerol trivalent gel. As discussed in section 7.2, this most likely arises due to an increase in the relaxation time of the polymer end-to-end vector.

7.1.2 Effect of Co-Solvent on Evaporative Endotherm

Given the low vapour pressure of dimethylformamide (DMF), 0.9-19 kPa from 300-370 K[38], in addition to its boiling point at 426.15 K it was imperative to determine the source of the evaporative endotherm being water derived.

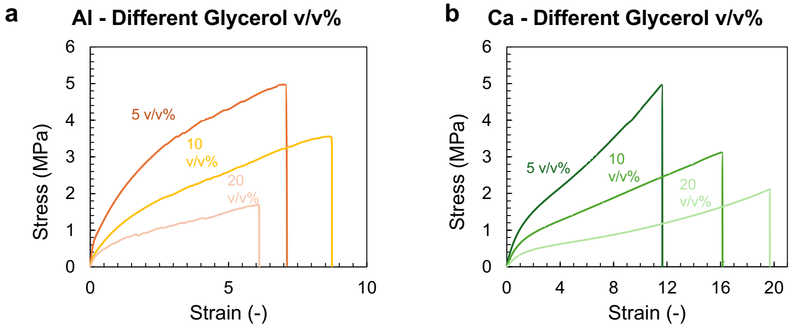

Sample	Modulus (MPa)	Std.Dev (MPa)
Al-H2O-5%	6.080	0.347
Al-H2O-10%	2.598	0.245
Al-H2O-20%	1.939	0.150

Sample	Modulus (MPa)	Std.Dev (MPa)
Ca-H2O-5%	2.139	0.157
Ca-H2O-10%	1.259	0.129
Ca-H2O-20%	0.467	0.034

Supplementary Fig. 39: Stress-strain curves obtained for different co-solvent contents on the trivalent (a) and divalent (b) MPEC gels. The respective storage moduli and standard deviations of each gel have been given in the tables above. The same protocol was used to print and perform the experiments on the gels as described in the main text.

The low mass loading of DMF in the system (<< 5% at maximum in resin) together with the vacuum pumping for 24 hours before equilibration at 30 mmHg is believed to have removed all residual DMF, suggesting the only reasonable evaporant remaining is the tightly bound waters coordinating the polymer and metal-carboxylate bonds. A simple experiment was designed to provide necessary and sufficient evidence of the water evaporation based origins this endotherm. The MPEC resins were reformulating to remove the need for DMF as co-solvent for the photo-initiator. DMF was only used as a co-solvent owing to the need to make miscible the TPO-L photoinitiator. We replaced the TPO-L photoinitiator with lithium phenyl (2,4,6-trimethylbenzoyl) phosphinate (LAP), another free radical photoinitiator with similar absorption wavelengths in the UV-violent range, thereby directly removing need of DMF. The resin was formulated in all other respects exactly identically to the recipe described in Table 1, save for substitution of DMF with water. Samples were photo-polymerized, pumped down, and equilibrated before DSC in the manner described in the main text.

As shown in Figure 40, samples showed similar exotherm-endotherm behavior consistent with prior results of Section 3.2 (Thermal Characterization). This observation was repeatable across multiple samples of various degree of hydration during the vacuum pump & equilibration process. The reduction in the magnitude of the endotherm after longer vacuum pumping is consistent with our understanding that the residual "loosely bound" solvent is pulled out of the gel. Therefore, this endotherm corresponds to the amount of solvent evaporated during this heating step; less solvent (water) corresponds to smaller

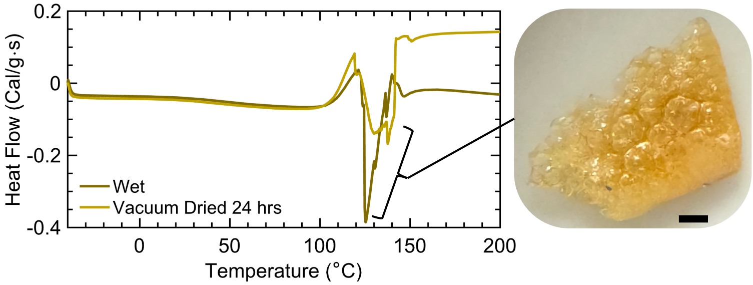

Supplementary Fig. 40: Dynamic scanning calorimetry of DMF-free Al^{3+}-MPEC gel as a representative species for the characteristic exotherm-endotherm signature found in MPEC gels in the vicinity of $\approx 120 - 160°C$ corresponding to desolvation and evaporation of tightly bound water. DSC performed at 5°C per minute from -40 to 400°C. Samples run at various states of hydration (wet from synthesis) and vacuum dried for 24 hours. Scale bar corresponds to 0.5 mm.

endotherm for a relatively similar sized exotherm. This confirms the observed thermal signature is characteristic of the MPEC gel and not a product of any residual DMF co-solvent that might persist in trace quantities.

7.2 Theoretical results

To probe the effect of the solvent on MPEC gels, we repeat the simulations performed in the main text. As an implicit solvent model was used to represent the system solvent, two parameters can be varied to account for changes in solvent: the dielectric constant of the system and the pressure applied. As there is a high concentration of polyelectrolyte, varying the solvent composition is not likely to have a dramatic effect on the system dielectric. On the other hand, by replacing glycerol with water, the overall free volume of the system is expected to decrease, leading to an increase in osmotic pressure. This effect is expected to be more substantial when varying the solvent. As such, we will observe the changes in the material behavior when varying the applied pressure. These results are summarised in figure 41.

As demonstrated in figure 41a, by increasing the system pressure (equivalent to increasing the water fraction in the solvent), the stress experienced at a particular deformation increases for all valencies. This is in good agreement with the experimental results from section 7.1. Examining figures 41b to 41d elucidates why this is the case. In general, figures 41b and 41d highlight that, through a reduction of the free volume of the system, the polymer motion has been greatly impeded, as demonstrated by an increased end-to-end vector relaxation time for all systems. This results in a narrower distribution of cluster sizes for the divalent and trivalent gels. Note that the polymer end-to-end vector autocorrelation time did not reach zero within the simulation time, making it difficult to quantitatively interpret the results in figure 41a for the

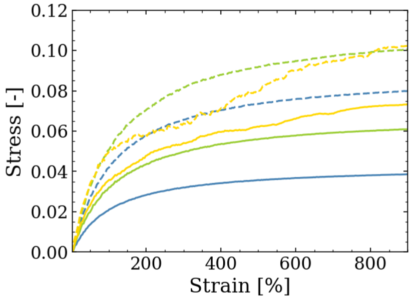

(a) Stress–Strain

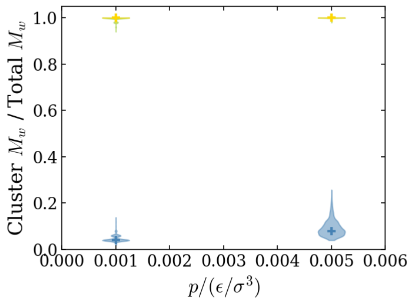

(b) Cluster Size

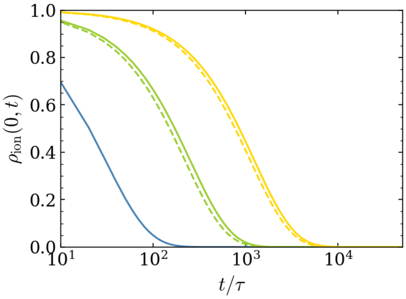

(c) Ion-pair relaxation

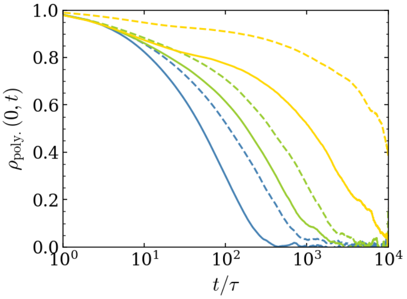

(d) End-to-end vector relaxation

Supplementary Fig. 41: Results obtained from molecular dynamics simulations of MPEC gels with varying applied pressure: **a,** Stress-strain curves from uniaxial stretching. **b,** Cluster size distribution under different system pressure. **c,** Ion-pair relaxation and **d,** end-to-end vector relaxation. Solid curves correspond to pressures of $0.001\epsilon/\sigma^3$ and dashed curves correspond to pressures of $0.005\epsilon/\sigma^3$. The color scheme used in this figure follow the scheme used in the main text (blue: monovalent, green: divalent and yellow: trivalent ions).

trivalent gel, beyond the qualitative increase in mechanical response. Longer simulations were not attempted as it appears that this system will require two orders of magnitude longer simulation time to fully equilibrate, something that was not computational feasible with the resources available to the authors at the time.

Interestingly, the monovalent gel observes a wider cluster distribution. This can be rationalized by the fact that, as clusters in the monovalent gels are not formed using dynamic crosslinks. As such, the reduced free volume brings the chains closer together, resulting in a larger average cluster size. Further, fluctuations in the cluster size will now arise from the motion of the individual chains. Given that the end-to-end vector relaxation time is still faster than the base divalent gels, these fluctuations will be more observable.

Another interesting observation is the limited impact of the change in free volume has not had a significant impact on the ion-pair relaxation times in figure 41c aside from a slight decrease. This decrease could be attributed to an increased proximity of the binding sites to each other as a result of the closer packing. However, the effect of the solvent is capable of influencing the polymer motion and mechanical behavior independently of the metal ion valency and system pH, giving an additional degree of flexibility.

8 Viscoelastic MPECs for controlling mechanical deformation

8.1 Strain-rate dependent buckling

We utilize the distinct mechanical signature of gels with different metal cations to demonstrate the synergetic interplay between the viscoelasticity of soft and highly deformable MPECs and the nonlinear mechanical response of a flexible and compliant structure.

Motivated by the recent work of strain-rate dependent mechanical mematerial, we followed the work of [39]. The visco-hyperelastic behavior of MPEC gels were modeled with the Neo-Hookean constants and Prony series. With the condition of incompressibility and uniaxial loading, the elastic moduli of the material in the loading direction is described as:

$$E(t) = 2E_0 \left(1 - \sum_{i=1}^{n} g_i (1 - e^{-\frac{t}{\tau_i}})\right) \left(2\lambda + \frac{1}{\lambda^2}\right) . \tag{S.56}$$

where n, E_o, g_i, τ_i are the number of series term, instantaneous modulus, coefficients of prony terms and time constants. For a bilayer construct, Al^{3+}-MPECs with high pH and Ni^{2+}-MPECs with low pH were selected for each layer. Stress relaxation experiments were conducted to measure the viscoelasticity of each material, and the relaxed modulus was fitted with prony series using lsqcurvefit algorithmas. As shown in Supplementary Fig. 42, a four-term prony series was fitted to determine the material parameters for each sample.

a

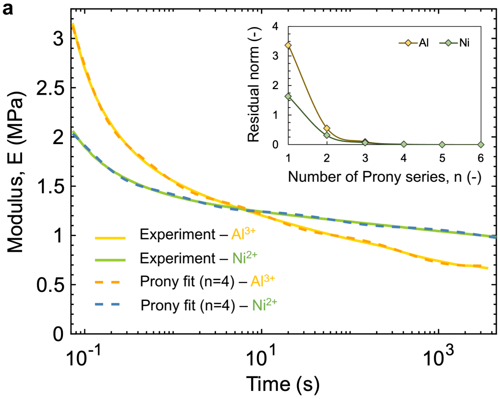

b

Prony – Al^{3+} at High pH

E_0^{Al} = 3.1286 MPa

g_i^{Al}(MPa)	τ_i^{Al}(s)
0.3172	0.0578
0.2216	0.5609
0.1253	12.0276
0.1149	417.7719

Prony – Ni^{2+} at Low pH

E_0^{Ni} = 2.0330 MPa

g_i^{Ni} (MPa)	τ_i^{Ni} (s)
0.2383	0.0989
0.1391	1.4919
0.0813	63.4001
0.0824	3320.2032

Supplementary Fig. 42: **a,** Fitting of experimental data of relaxation modulus for Al^{3+} at high pH (yellow) and Ni^{2+} at low pH (green). A four-term Prony series (n=4) was selected for fitting based on the residual norm analysis (inset). **b,** The fitting parameters determined for each material.

To model the buckling criteria, the Euler buckling conditions for a finite beam on elastic foundation were used [40]. The implicit equation for the buckling was given as:

$$\frac{\sin(\frac{1}{2}\sqrt{\pi^2 x + 2y})}{\sin(\frac{1}{2}\sqrt{\pi^2 x - 2y})} = \mp \frac{\sqrt{\pi^2 x + 2y}}{\sqrt{\pi^2 x - 2y}} \tag{S.57}$$

$$\text{where } x = \frac{P_{cr} l^2}{\pi^2 EI} \text{ and } y = \sqrt{\frac{k_f l^4}{EI}}.$$

which simplifies into an analytical solution for the critical buckling load, P_{cr} and/or critical buckling strain, ε_{cr} is given as:

$$\varepsilon_{cr} = \frac{P_{cr}}{EA} = \frac{2I}{l^2 A}\left(\sqrt{\frac{k_f l^4}{EI}} + 2\pi^2\right) \tag{S.58}$$

where E, k_f, l, I, A are elastic modulus of the beam, stiffness of the elastic foundation, effective length of buckling, and the moment of inertia, and the cross section area of a beam. For the same geometry of each layer on the beam ($l, I,$ and$I$ are identical for both materials), the buckling criteria for the bilayer construct towards the side of Al^{3+}-MPEC can be written as:

$$\varepsilon_{cr,Al} > \varepsilon_{cr,Ni} \tag{S.59}$$

$$\sqrt{\frac{E_{Ni}l^4}{E_{Al}I}} > \sqrt{\frac{E_{Al}l^4}{E_{Ni}I}} \tag{S.60}$$

$$E_{Ni} > E_{Al} \tag{S.61}$$

Combining with the visco-hyperelastic model (equation S.56), the criterion – buckling towards the side of Al^{3+}-MPEC – can be given as:

$$\frac{E_{0,Ni}}{E_{0,Al}} > \frac{\left(1 - \sum_{i=1}^{n} g_{i,Al}(1 - e^{-\frac{t}{\tau_{i,Al}}})\right)}{\left(1 - \sum_{i=1}^{n} g_{i,Ni}(1 - e^{-\frac{t}{\tau_{i,Ni}}}\right)} \tag{S.62}$$

For the lower ($t \to \infty$) and higher ($t \to 0$) limits of strain rates, equation S.62 can be simplified as

$$\frac{E_{0,Ni}}{E_{0,Al}} > \frac{(1 - \sum_{i=1}^{n} g_{i,Al}))}{(1 - \sum_{i=1}^{n} g_{i,Ni})} \text{ for lower strain rate } (t \to \infty) \tag{S.63}$$

$$\frac{E_{0,Ni}}{E_{0,Al}} > 1 \text{ for high strain rate } (t \to 0) \tag{S.64}$$

Uniaxial compression and tension experiments conducted on the bilayer beam of Al^{3+}-Ni^{2+} (left-right side) at a strain rate of $10^{-3}s^{-1}$ (slow loading) and $10^{-1}s^{-1}$ (fast loading) to demonstrate the strong dependence of instability deformation mode on material compositions and applied strain rate.

References

[1] Rubinstein, M., Colby, R.H.: Polymer Physics. Oxford University Press, Oxford, New York (2003)

[2] Taylor, T.J., Stivala, S.S.: Small-angle X-ray scattering of poly(acrylic acid) in solution: 1. Dioxane. Polymer **37**(5), 715–719 (1996). https://doi.org/10.1016/0032-3861(96)87245-4. Accessed 2023-10-16

[3] Amthauer, G., Annerste, H., Hafner, S.: The mössbauer spectrum of 57fe in silicate garnets. Zeitschrift für Kristallographie-Crystalline Materials **143**(1-6), 14–55 (1976)

[4] Sipos, P., Zeller, D., Kuzmann, E., Vértes, A., Homonnay, Z., Walczak, M., Canton, S.E.: The structure of fe (iii) ions in strongly alkaline aqueous solutions from exafs and mössbauer spectroscopy. Dalton Transactions (41), 5603–5611 (2008)

[5] Garvie, L.A., Buseck, P.R.: Ratios of ferrous to ferric iron from nanometre-sized areas in minerals. Nature **396**(6712), 667–670 (1998)

[6] Fultz, B.: Mössbauer Spectrometry. In: Characterization of Materials. John Wiley, New York (2011)

[7] Blaško, M., Pašteka, L.F., Urban, M.: DFT Functionals for Modeling of Polyethylene Chains Cross-Linked by Metal Atoms. DLPNO–CCSD(T) Benchmark Calculations. The Journal of Physical Chemistry A **125**(34), 7382–7395 (2021). https://doi.org/10.1021/acs.jpca.1c04793

[8] Becke, A.D.: A new mixing of Hartree–Fock and local density-functional theories. The Journal of Chemical Physics **98**(2), 1372–1377 (1993). https://doi.org/10.1063/1.464304

[9] Grimme, S., Antony, J., Ehrlich, S., Krieg, H.: A consistent and accurate ab initio parametrization of density functional dispersion correction (DFT-D) for the 94 elements H-Pu. J. Chem. Phys. **132**(15), 154104 (2010). https://doi.org/10.1063/1.3382344

[10] Weigend, F., Ahlrichs, R.: Balanced basis sets of split valence, triple zeta valence and quadruple zeta valence quality for H to Rn: Design and assessment of accuracy. Physical Chemistry Chemical Physics **7**(18), 3297–3305 (2005). https://doi.org/10.1039/B508541A

[11] Weigend, F.: Accurate Coulomb-fitting basis sets for H to Rn. Physical Chemistry Chemical Physics **8**(9), 1057–1065 (2006). https://doi.org/10.1039/B515623H

[12] Mennucci, B., Tomasi, J.: Continuum solvation models: A new approach to the problem of solute's charge distribution and cavity boundaries. The Journal of Chemical Physics **106**(12), 5151–5158 (1997). https://doi.org/10.1063/1.473558

[13] Chai, J.-D., Head-Gordon, M.: Systematic optimization of long-range corrected hybrid density functionals. The Journal of Chemical Physics **128**(8), 084106 (2008). https://doi.org/10.1063/1.2834918

[14] Grimme, S., Ehrlich, S., Goerigk, L.: Effect of the damping function in dispersion corrected density functional theory. Journal of Computational Chemistry **32**(7), 1456–1465 (2011). https://doi.org/10.1002/jcc.21759

[15] Abrahamson, H.B., Rezvani, A.B., Brushmiller, J.G.: Photochemical and spectroscopic studies of complexes, of iron (iii) with citric acid and other carboxylic acids. Inorganica Chimica Acta **226**(1-2), 117–127 (1994)

[16] Kuma, K., Nakabayashi, S., Suzuki, Y., Kudo, I., Matsunaga, K.: Photoreduction of fe (iii) by dissolved organic substances and existence of fe (ii) in seawater during spring blooms. Marine Chemistry **37**(1-2), 15–27 (1992)

[17] Lueder, U., Jørgensen, B.B., Kappler, A., Schmidt, C.: Photochemistry of iron in aquatic environments. Environmental Science: Processes & Impacts **22**(1), 12–24 (2020)

[18] Landsgesell, J., Nová, L., Rud, O., Uhlík, F., Sean, D., Hebbeker, P., Holm, C., Košovan, P.: Simulations of ionization equilibria in weak polyelectrolyte solutions and gels. Soft Matter **15**(6), 1155–1185 (2019). https://doi.org/10.1039/C8SM02085J

[19] Landsgesell, J., Hebbeker, P., Rud, O., Lunkad, R., Košovan, P., Holm, C.: Grand-Reaction Method for Simulations of Ionization Equilibria Coupled to Ion Partitioning. Macromolecules **53**(8), 3007–3020 (2020). https://doi.org/10.1021/acs.macromol.0c00260

[20] Deacon, G.B., Phillips, R.J.: Relationships between the carbon-oxygen stretching frequencies of carboxylato complexes and the type of carboxylate coordination. Coordination Chemistry Reviews **33**(3), 227–250 (1980). https://doi.org/10.1016/S0010-8545(00)80455-5. Accessed 2023-10-15

[21] Deacon, G.B., Huber, F., Phillips, R.J.: Diagnosis of the nature of carboxylate coordination from the direction of shifts of carbon-oxygen stretching frequencies. Inorganica Chimica Acta **104**(1), 41–45 (1985). https://doi.org/10.1016/S0020-1693(00)83783-4. Accessed 2023-10-15

[22] Kirwan, L.J., Fawell, P.D., van Bronswijk, W.: In Situ FTIR-ATR Examination of Poly(acrylic acid) Adsorbed onto Hematite at Low pH. Langmuir **19**(14), 5802–5807 (2003). https://doi.org/10.1021/la027012d. Publisher: American Chemical Society. Accessed 2023-05-05

[23] Boisvert, J.-P., Malgat, A., Pochard, I., Daneault, C.: Influence of the counter-ion on the effective charge of polyacrylic acid in dilute condition. Polymer **43**(1), 141–148 (2002). https://doi.org/10.1016/S0032-3861(01)00603-6. Accessed 2023-10-15

[24] Wojdyr, M.: Fityk: a general-purpose peak fitting program. Journal of Applied Crystallography **43**(5), 1126–1128 (2010). https://doi.org/10.1107/S0021889810030499. Number: ARRAY(0xacfea08) Publisher: International Union of Crystallography. Accessed 2023-10-18

[25] Chapman, W.G., Gubbins, K.E., Jackson, G., Radosz, M.: SAFT: Equation-of-state solution model for associating fluids. Fluid Phase Equilib. **52**, 31–38 (1989). https://doi.org/10.1016/0378-3812(89)80308-5

[26] Carnahan, N.F., Starling, K.E.: Equation of State for Nonattracting Rigid Spheres. The Journal of Chemical Physics **51**(2), 635–636 (2003). https://doi.org/10.1063/1.1672048

[27] Zhang, P., Alsaifi, N.M., Wu, J., Wang, Z.-G.: Salting-Out and Salting-In of Polyelectrolyte Solutions: A Liquid-State Theory Study. Macromolecules **49**(24), 9720–9730 (2016). https://doi.org/10.1021/acs.macromol.6b02160

[28] Debye, P., Hückel, E.: Zur Theorie der Elektrolyte. I. Gefrierpunktserniedrigung und verwandte Erscheinungen. Physikalische Zeitschrift **24**, 185–206 (1923)

[29] Silva, W., Zanatta, M., Ferreira, A.S., Corvo, M.C., Cabrita, E.J.: Revisiting Ionic Liquid Structure-Property Relationship: A Critical Analysis. International Journal of Molecular Sciences **21**(20), 7745 (2020). https://doi.org/10.3390/ijms21207745

[30] Ylitalo, A.S., Balzer, C., Zhang, P., Wang, Z.-G.: Electrostatic Correlations and Temperature-Dependent Dielectric Constant Can Model LCST in Polyelectrolyte Complex Coacervation. Macromolecules **54**(24), 11326–11337 (2021). https://doi.org/10.1021/acs.macromol.1c02000

[31] Blum, L.: Mean spherical model for asymmetric electrolytes. Molecular Physics **30**(5), 1529–1535 (1975). https://doi.org/10.1080/00268977500103051

[32] Maribo-Mogensen, B., Kontogeorgis, G.M., Thomsen, K.: Modeling of

Dielectric Properties of Complex Fluids with an Equation of State. The Journal of Physical Chemistry B **117**(12), 3389–3397 (2013). https://doi.org/10.1021/jp310572q

[33] Semenov, A.N., Rubinstein, M.: Thermoreversible Gelation in Solutions of Associative Polymers. 1. Statics. Macromolecules **31**(4), 1373–1385 (1998). https://doi.org/10.1021/ma970616h

[34] Blum, L., Hoeye, J.S.: Mean spherical model for asymmetric electrolytes. 2. Thermodynamic properties and the pair correlation function. The Journal of Physical Chemistry **81**(13), 1311–1316 (1977). https://doi.org/10.1021/j100528a019

[35] Jiang, J.W., Blum, L., Bernard, O., Prausnitz, J.M.: Thermodynamic properties and phase equilibria of charged hard sphere chain model for polyelectrolyte solutions. Molecular Physics **99**(13), 1121–1128 (2001). https://doi.org/10.1080/00268970110043414

[36] Dufal, S., Lafitte, T., Haslam, A.J., Galindo, A., Clark, G.N.I., Vega, C., Jackson, G.: The A in SAFT: Developing the contribution of association to the Helmholtz free energy within a Wertheim TPT1 treatment of generic Mie fluids. Mol. Phys. **113**(9-10), 948–984 (2015). https://doi.org/10.1080/00268976.2015.1029027

[37] Gross, J., Sadowski, G.: Application of the Perturbed-Chain SAFT Equation of State to Associating Systems. Ind. Eng. Chem. Res. **41**(22), 5510–5515 (2002). https://doi.org/10.1021/ie010954d

[38] Cui, X., Chen, G., Han, X.: Experimental Vapor Pressure Data and a Vapor Pressure Equation for N,N-Dimethylformamide. Journal of Chemical & Engineering Data **51**(5), 1860–1861 (2006). https://doi.org/10.1021/je060224i. Publisher: American Chemical Society. Accessed 2024-05-29

[39] Janbaz, S., Narooei, K., van Manen, T., Zadpoor, A.A.: Strain rate–dependent mechanical metamaterials. Science Advances **6**(25), 0616 (2020). https://doi.org/10.1126/sciadv.aba0616. Publisher: American Association for the Advancement of Science. Accessed 2023-10-14

[40] Hetényi, M.: Beams on Elastic Foundation: Theory with Applications in the Fields of Civil and Mechanical Engineering. University of Michigan Press, Ann Habor, Michigan (1946)
